# Relevance of diet in schizophrenia: a review focusing on prenatal nutritional deficiency, obesity, oxidative stress and inflammation

**DOI:** 10.3389/fnut.2024.1497569

**Published:** 2024-12-13

**Authors:** Viorica Rarinca, Amalia Vasile, Malina Visternicu, Vasile Burlui, Gabriela Halitchi, Alin Ciobica, Ana-Maria Singeap, Romeo Dobrin, Ecaterina Burlui, Lucian Maftei, Anca Trifan

**Affiliations:** ^1^Doctoral School of Geosciences, Faculty of Geography and Geology, Alexandru Ioan Cuza University of Iasi, Iași, Romania; ^2^Doctoral School of Biology, Faculty of Biology, Alexandru Ioan Cuza University of Iași, Iași, Romania; ^3^Preclinical Department, Apollonia University, Iași, Romania; ^4^Faculty of Biology, “Alexandru Ioan Cuza” University of Iași, Iași, Romania; ^5^CENEMED Platform for Interdisciplinary Research, “Grigore T. Popa” University of Medicine and Pharmacy of Iasi, Iași, Romania; ^6^Romanian Academy of Scientists, Bucharest, Romania; ^7^Department of Gastroenterology, “Grigore T. Popa” University of Medicine and Pharmacy, Iași, Romania; ^8^Institute of Gastroenterology and Hepatology, “Sf. Spiridon”, Iași, Romania; ^9^“Socola” Psychiatric Institute, Iași, Romania; ^10^“Grigore T. Popa” University of Medicine and Pharmacy, Iași, Romania; ^11^SC MAKEUP SHOP SRL – Cosmetics Product Development Department, Iași, Romania

**Keywords:** schizophrenia, diet, nutrients, obesity, oxidative stress, inflammation, antioxidants, prenatal nutritional deficiency justified

## Abstract

**Background/Objectives:**

Schizophrenia is a complex mental disorder influenced by genetic and environmental factors, including dietary habits. Oxidative stress and inflammation play a crucial role in the pathophysiology of schizophrenia. Emerging research suggests that diet may affect schizophrenia through different biological mechanisms beyond oxidative stress and inflammation. In particular, epigenetic changes may alter the expression of genes related to neurodevelopment and neurotransmitter systems, while neuroplasticity plays a crucial role in brain adaptation and resilience to psychiatric disorders.

**Methods:**

The literature search included the main available databases (Science Direct, PubMed and Google Scholar), considering the English language, and our screening was performed based on several words such as “schizophrenia”, “diet”, “nutrients”, “obesity”, “oxidative stress”, “inflammation”, “antioxidants” and “prenatal nutritional deficiency”. The review focused specifically on studies examining the relevance of diet in schizophrenia, as well as prenatal nutritional deficiency, obesity, oxidative stress, and inflammation associated with this disorder.

**Results:**

Following a review of the literature, it was found that nutritional deficiencies, including lack of omega-3 fatty acids, vitamins D, and B, during the prenatal and postnatal periods can have a negative impact on neurodevelopment and increase the risk of schizophrenia. Patients with schizophrenia have imbalances in antioxidant enzymes, such as glutathione peroxidase (GPx), superoxide dismutase (SOD), catalase (CAT), and reduced levels of antioxidants (vitamin E, vitamin C). These biochemical changes lead to an increase in markers of oxidative stress, including malondialdehyde (MDA). In addition, cytokine-mediated inflammation, microglial activation, and intestinal dysbiosis are associated with the onset of schizophrenia and the severity of schizophrenia symptoms. Currently, there is no universally accepted dietary regimen for control. However, various diets and nutritional methods are being researched and applied to alleviate the symptoms of schizophrenia and improve the overall health of patients, including the Mediterranean diet, the ketogenic diet, the gluten-free diet, and the DASH (Dietary Approaches to Stop Hypertension) diet.

**Conclusion:**

A healthy diet, rich in anti-inflammatory nutrients and antioxidants, may help manage schizophrenia by reducing oxidative stress, preventing complications, and improving quality of life. Omega-3 fatty acids, vitamin D, and B vitamins are particularly important for brain development and function. In this review, we aim to analyze the literature on the influence of diet on schizophrenia, focusing on the role of prenatal nutritional deficiencies, obesity, oxidative stress, and inflammation.

## 1 Introduction

Common mental disorders such as schizophrenia, depression, and anxiety are major public health concerns worldwide. These conditions currently represent the greatest challenge to disability in developed countries, severely limiting the capacity for daily functioning. Poor nutrition, lack of physical exercise, and smoking have long been recognized as primary contributors to common non-communicable diseases such as heart disease, type 2 diabetes mellitus, and cancer (1). Psychiatric patients are at higher risk of premature mortality from cardiovascular disease. Psychiatric conditions are characterized by an increased risk of metabolic syndrome (MetS), grouping several cardiovascular risk factors, including obesity, dyslipidemia, hyperglycemia, and hypertension. They are at risk for several psychiatric conditions, including anxiety, major depressive disorder, attention deficit hyperactivity disorder, and schizophrenia (2).

Schizophrenia is a chronic, complex, and debilitating mental disorder that involves issues with cognition, mood, memory, reality perception, and interpersonal relationships (3, 4). The literature suggests that schizophrenia can result from a combination of genetic and environmental factors, including stress, diet, lack of physical activity, and medication (5). Diagnosing schizophrenia requires specialized professionals to identify a complex group of symptoms, which include cognitive impairments, disorganization, and negative and positive symptoms. These signs and symptoms are clearly defined in international disease classifications such as the Diagnostic and Statistical Manual of Mental Disorders (DSM) and the International Classification of Diseases (ICD) (6).

**Figure 1:**
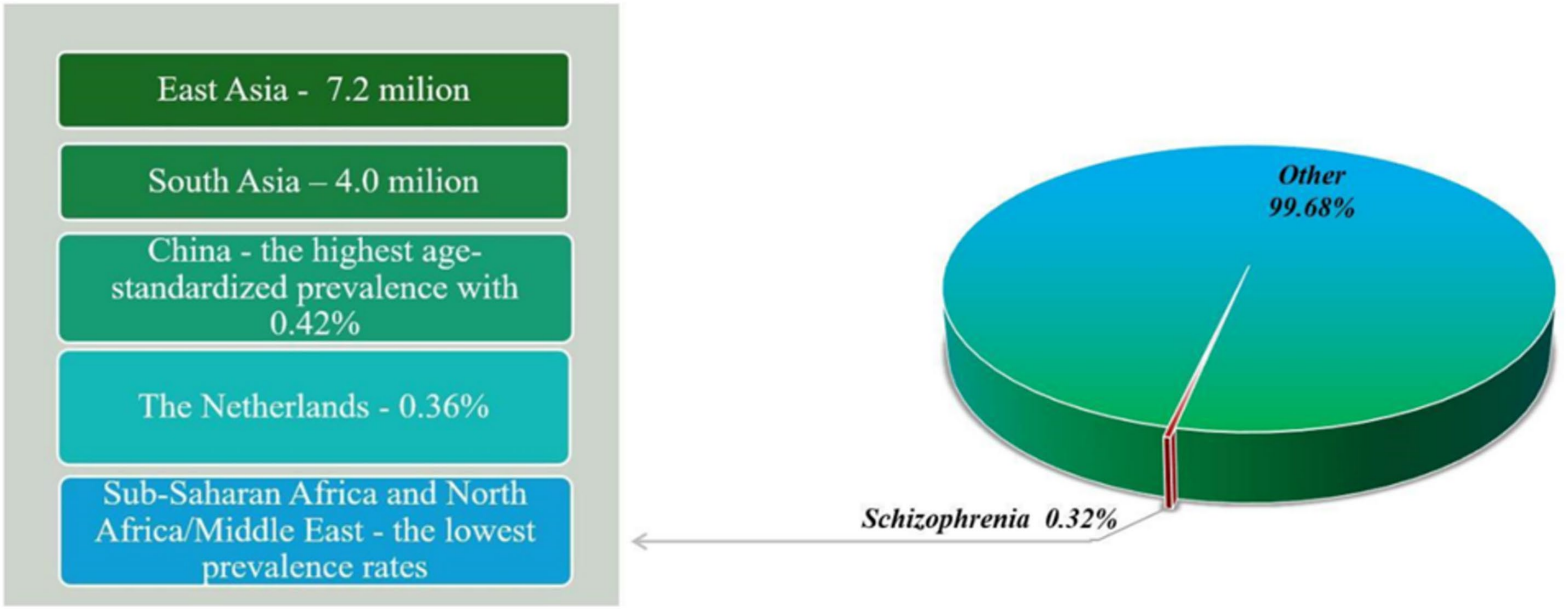
Prevalence of schizophrenia in different regions in 2019.

Schizophrenia is a serious mental disorder that affects approximately 1% of the global population (7). According to the World Health Organization (WHO), schizophrenia affects approximately 24 billion people, a percentage of 0.32% worldwide. Although not as common as other mental disorders, it affects 0.45% of adults (WHO). The estimated age-standardized prevalence of schizophrenia is 0.28% (95% UI: 0.24–0.31) (8). In 2019, 23.6 million people were living with schizophrenia worldwide, an increase of 65.85% since 1990 (9). The prevalence of schizophrenia varies by region and country, with East Asia and South Asia having the highest number of cases, with approximately 7.2 million and 4.0 million cases, respectively, in 2016 (Figure 1) (8). While China has the highest age-standardized prevalence with 0.42% (95% UI: 0.38–0.48), the Netherlands has a higher prevalence than other Western European countries, at 0.36% (95% UI: 0.32–0.40) (8). On the other hand, some of the lowest prevalence rates are found in sub-Saharan Africa and North Africa/Middle East (8). People with schizophrenia have a significantly reduced life expectancy compared to the general population, so studies have shown that people with schizophrenia die 10–25 years earlier than the general population (10).

Although defined by the presence of psychotic symptoms, schizophrenia remains both etiologically and therapeutically challenging, being more common in males than females (11). People with schizophrenia have a 2 to 5 times higher risk of developing type 2 diabetes compared to the general population (12). Imbalances in neurotransmission have been the basis of many theories regarding the pathophysiology of schizophrenia. These theories focus on chemical imbalances in the brain, either through excess or deficiency of neurotransmitters such as dopamine, serotonin, and glutamate. Other theories suggest the involvement of substances such as glycine, aspartate and gamma-aminobutyric acid (GABA) in the neurochemical imbalance specific to schizophrenia (13).

Due to the complexity of the causes of schizophrenia, current antipsychotic treatments focus mainly on symptom relief (14). Clozapine is considered the most effective antipsychotic for treating schizophrenia, with a success rate of approximately 30% in controlling episodes in patients resistant to other treatments, compared to only 4% for the combination of chlorpromazine and benztropine. Clozapine has also been associated with increased serum sodium concentrations in patients with polydipsia and hyponatremia (13).

In the field of psychiatry, nutritional interventions have been much studied in recent times, with an emphasis on nutrient supplementation and the adoption of specific dietary strategies to prevent and treat mental disorders (15). The field of ‘nutritional psychiatry’ has developed rapidly in recent years, with several studies related to dietary or nutrient-based interventions being initiated, and more preclinical and epidemiological data becoming available (16). The last 10 years have seen a steady increase in research examining the links between nutrition and mental health. Various studies have confirmed that nutrition is a factor preceding the onset of psychiatric symptoms, indicating a direction of causality (17). In addition to drug treatments, the importance of nutrition in mental health is increasingly recognized. Nutritional deficiencies or excesses have been identified as significant factors in influencing mental health. Recently, nutritional interventions have been explored not only for high-prevalence mental disorders such as depression and anxiety but also for rarer disorders such as schizophrenia (3).

An increasing number of studies suggest that certain modifiable lifestyle behaviors are also risk factors for common mental disorders. The quality of diet has become a primary focus in recent research concerning lifestyle and mental health (1). Previous studies have established a strong connection between diet, cognitive functions, and mental health. For example, a systematic review analyzing various dietary patterns and cognitive functions showed that increased carbohydrate intake (such as sugars) is linked to a decline in overall cognitive performance, while saturated fats were associated with reduced memory and learning. Conversely, protein intake has been observed to positively impact executive function and memory (18). Recent studies state that dietary intake during pregnancy influences maternal health, and poor dietary habits during pregnancy produce various complications (19, 20). Fetal brain development can be particularly vulnerable to factors such as maternal nutrition, infection and stress during pregnancy. Both overnutrition and undernutrition can have consequences on fetal neurodevelopment (21). Inadequate nutrition in the first trimester of pregnancy affects the fetus with negative consequences for it (19). Among the negative consequences is the risk of offspring schizophrenia through epigenetic effects (22).

Diet and mental health are connected through changes in molecular biomarkers as well as alterations in brain structure and function. Nutrition studies indicate that the relationship between eating habits and mental disorders (or cognitive ability) may be influenced by gut-brain communication (18). In recent years, researchers have focused on the role of gut microbiota on diet. The gut microbiota has a critical role in mediating the effects of nutritional deficiencies on neurotransmitters and affects brain functions through multiple neural, endocrine and immune pathways. Its immunomodulatory mechanisms can produce both anti-inflammatory and pro-inflammatory effects, impacting not only the central nervous system but also the entire body. These effects are mediated by regulation of pro-inflammatory and anti-inflammatory cytokine levels and modulation of glial functions. In addition, the microbiota plays a role in maintaining oral tolerance, controlling intestinal and blood–brain barrier permeability, and modulating the hypothalamic–pituitary–adrenal axis, which in turn influences tryptophan metabolism. Gut microbes are the source of various neuroactive substances and neurotransmitters, including short-chain fatty acids, GABA, norepinephrine, dopamine, histamine, serotonin, and acetylcholine (23). In addition to their role in food digestion, metabolism, and fat storage, gut microbes have been observed to influence neurotransmitter balance (3). A growing body of evidence points to the significant impact of the gut microbiome on the development and progression of obesity. Dysbiosis, characterized by reduced diversity and changes in the composition of the gut microbiome, is associated with overweight and obesity (5). Another recent trend in nutritional psychiatry involves research examining the influence of modulating the gut microbiota through dietary interventions and probiotic and prebiotic supplements in treating various neuropsychiatric conditions (17).

In addition, António Damásio argues that the gut should be viewed as “our first brain” from an evolutionary point of view, emphasizing its essential role in emotional and cognitive processes. He hypothesizes that early life forms were primarily “floating digestive tracts,” where the main function of the nervous system was to regulate digestion. As organisms evolved, this nervous system expanded to manage various body functions, leading to a complex network of neurons around the gut that influence emotional regulation and feelings. Damasio emphasizes the gut-brain connection, noting that the enteric nervous system communicates with the brain and is integral to our emotional experiences. He asserts that bodily states, such as hunger and pain, are fundamental to consciousness, linking physical sensations to mental experiences (24).

The general public’s diet falls significantly short of the standards recommended by the WHO for a healthy diet. Young people, who are at the highest risk for developing schizophrenia, tend to have particularly poor diets, often consuming large quantities of fast food. Research indicates that individuals with schizophrenia have even more inadequate diets compared to the general population (25). They exhibit an excessive preference for unhealthy foods, especially fast food and foods high in fats and carbohydrates, which characterize their dietary habits (26). This review aims to critically examine the evidence for the influence of diet, with a focus on prenatal nutritional deficiencies, obesity, oxidative stress, and inflammation, on the development, progression, and severity of schizophrenia symptoms. This review employs the PICOS framework to systematically evaluate the influence of diet on schizophrenia, providing a structured approach to understanding the relevant literature.

## 2 Methodology

### 2.1 Search strategy

The current systematic review was conducted following the Preferred Reporting Items for Systematic Reviews and Meta-Analysis (PRISMA) guidelines, employing several electronic databases (Science Direct, PubMed, and Google Scholar) and using the following keywords: [(schizophrenia[Title/Abstract]) AND (Diet [Title/Abstract]) AND (nutrients [Title/Abstract]) AND (obesity [Title/Abstract]) AND (oxidative stress [Title/Abstract]) AND (inflammation [Title/Abstract]) AND (antioxidants [Title/Abstract])]. Inclusion criteria included studies until July 2024 in English, which evaluates the relevance of diet in schizophrenia focusing on prenatal nutritional deficiency, obesity, oxidative stress and inflammation.

### 2.2 PICOS framework

**Figure 2:**
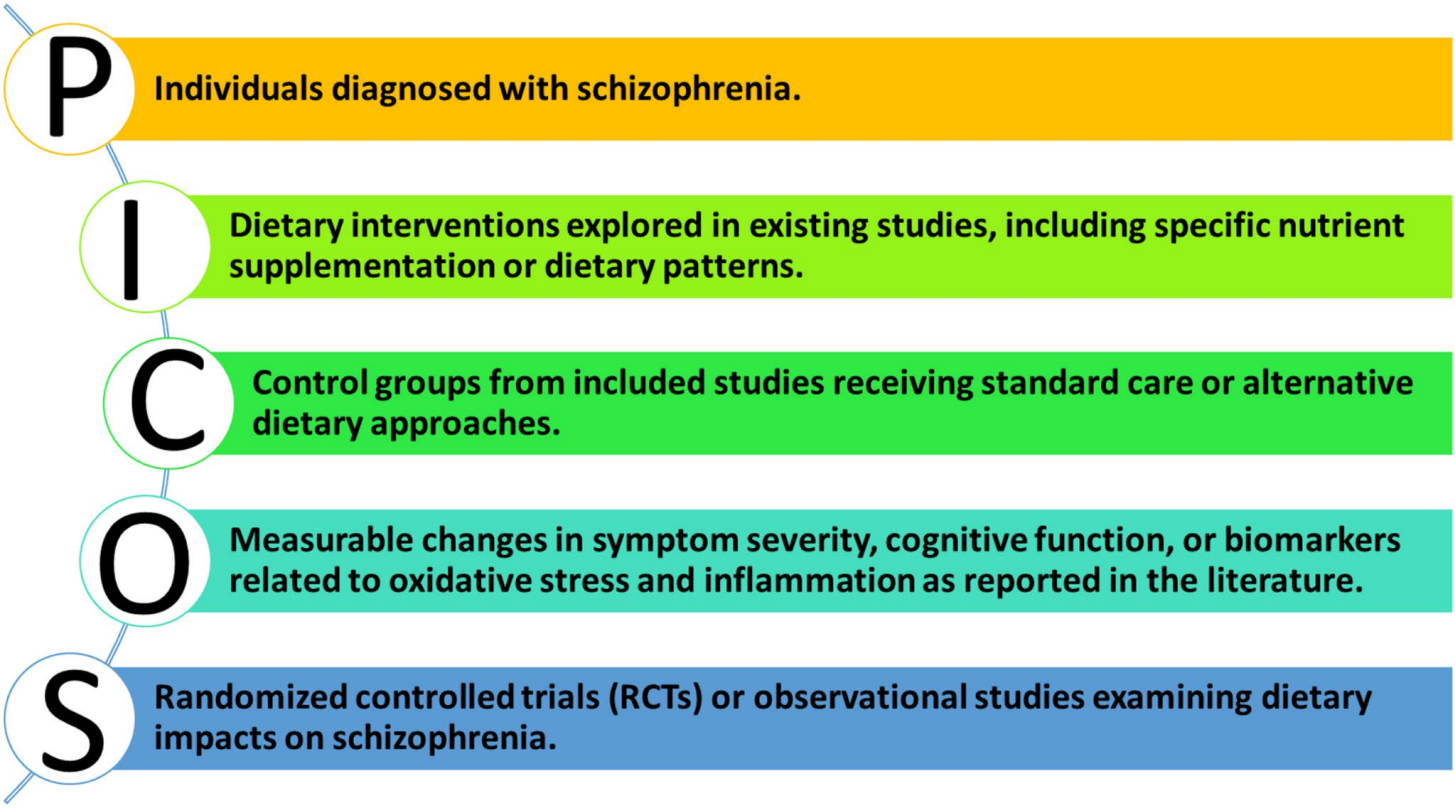
PICOS framework for evaluating dietary influences on schizophrenia.

To ensure a systematic approach to our review, we employed the PICOS framework as illustrated in Figure 2.

### 2.3 Exclusion criteria

We applied the following exclusion criteria: (1) case reports, letters, summaries, expert opinion and comments; (2) conference abstracts, books, book chapters, and unpublished results; (3) non-English papers.

### 2.4 Data extraction

Among the initial 1,324 reports that were collected through electronic search, 878 were omitted due to duplicated results, 273 were ruled out because of the article type, and an additional 209 were excluded as they comprised conference abstracts, books, book chapters, and unpublished results. Additionally, 8 was excluded because it was not in the English language.

### 2.5 Data synthesis

**Figure 3:**
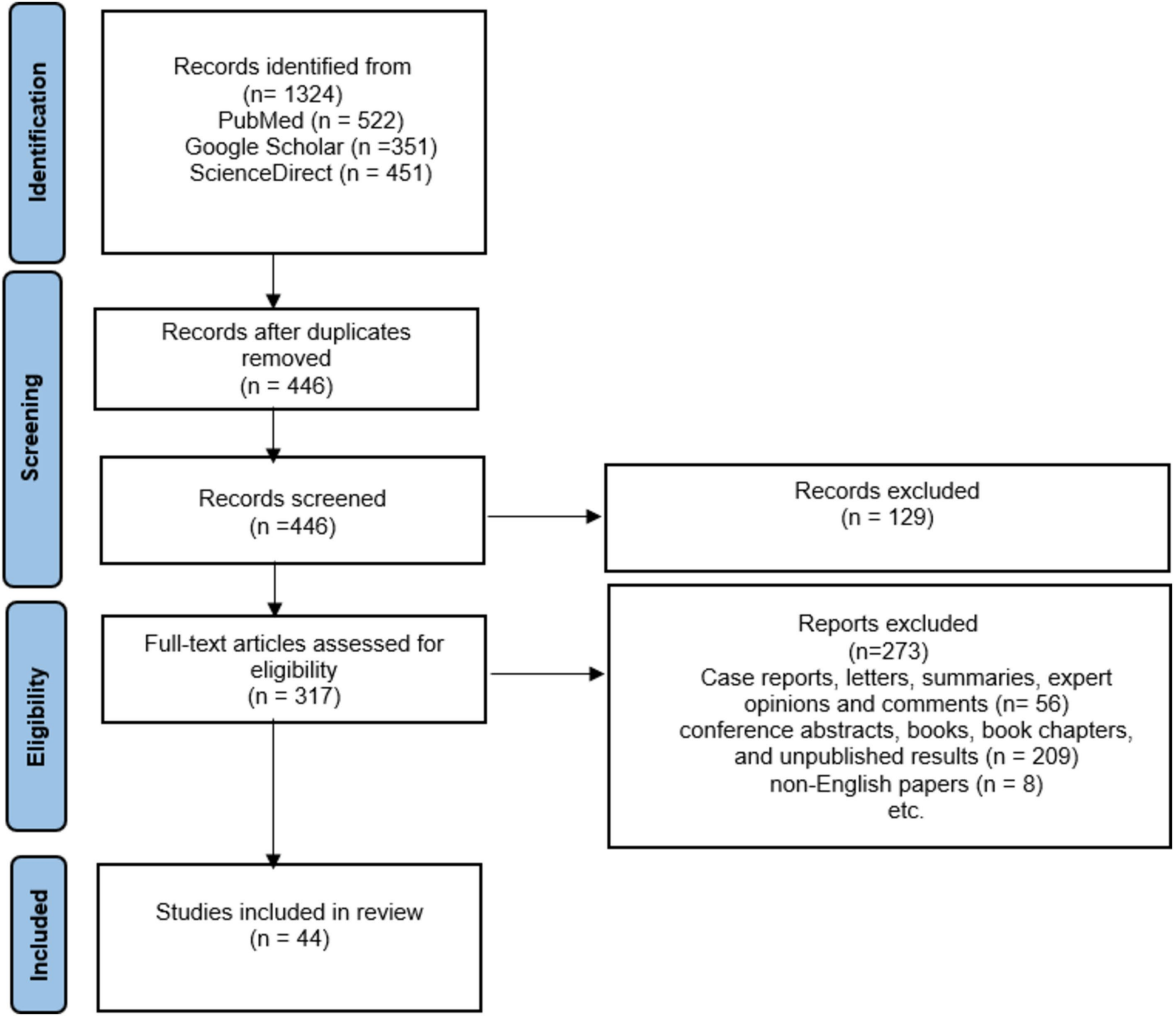
The PRISMA flow chart of the selection process for the included studies.

Finally, 44 articles were included in this study, as demonstrated in a diagram of the literature search and selection process (see Figure 3). It was thought that the studies would be too heterogeneous to be combined. Therefore, a narrative synthesis was performed. The results are summarized in three chapters that address deficiencies in prenatal nutrition, obesity in schizophrenia and the role of diet and antidepressants, but also the impact of diet on oxidative stress and inflammation in schizophrenia.

## 3 Impact of prenatal nutritional deficiencies on schizophrenia risk

Increasing evidence from nutritional psychiatry shows that diet is crucial for psychological well-being throughout life. Recently, nutrition has become essential in modifying brain functions and their plasticity, as there is a connection between poor childhood nutrition and an increased risk of developing psychiatric disorders later in life (27). For example, folic acid deficiency during prenatal and postnatal periods can negatively affect neurological development and increase the likelihood of developing mental health issues (28). Deficiency of several nutrients, such as essential fatty acids, retinoids, vitamin D, and iron, has been associated with an increased risk of schizophrenia due to malnutrition during pregnancy (29).

Maternal immune activation (MIA) during pregnancy has been increasingly recognized as a significant factor influencing fetal neurodevelopment, particularly about the risk of schizophrenia in offspring. Research indicates that prenatal infections can trigger MIA, leading to elevated levels of pro-inflammatory cytokines that may disrupt neurodevelopmental processes.

A comprehensive review by Brown and Derkits (30), which included epidemiological and translational studies, showed that there is a correlation between prenatal infections and an increased risk of schizophrenia in offspring. These findings underscore the importance of maternal health during pregnancy, particularly about infections that can provoke immune responses. The mechanisms by which MIA can disrupt neurodevelopment were also studied by Kwon et al. (31), thus highlighting both experimental models and clinical evidence showing that maternal immune activation can lead to long-lasting changes in brain structure and function associated with an increased risk of developing schizophrenia. These changes may include changes in neurotransmitter systems, particularly those involving dopamine, which is essential for cognitive and emotional regulation.

In addition, the Gut-Brain Axis plays a crucial role in mediating the effects of maternal diet on neurodevelopment and schizophrenia risk. Thus, Cryan et al. (32) detailed how the gut microbiome can influence brain function through various mechanisms, including modulation of inflammation, neurotransmitter synthesis, and stress response. This connection suggests that dietary factors affecting gut microbiota composition could indirectly impact neurodevelopment through inflammatory pathways.

As some authors state, the intestinal microbiome plays an essential role in both the mother’s and the newborn’s diet, because the composition of the maternal microbiome is related to a child’s risk of developing certain adverse events such as certain diseases (33, 34). Moreover, the infant microbiome impacts neonatal development in several aspects, including the development of metabolism, neural, and immune responses. Disruption of gut microbiota composition in the first years of life may increase the risk of food intolerance and necrotizing enterocolitis (34). For the establishment of the gut microbiota, breastfeeding is an important period because the profile of the infant’s gut microbiome is time-dependent, with different types of microbes at different stages of early development. It has been found that cesarean delivery, antibiotic exposure, or formula feeding can disrupt the gut microbiota, for example, Lactobacillus and Bifidobacterium are more abundant in breastfed infants compared to formula-fed infants (34).

Furthermore, preterm infants have a different gut microbial community than full-term infants. Several factors can influence the colonization, development, and function of the gut microbiota of preterm infants (34, 35). Maternal microbiota and infant microbiota can be affected by genetic and environmental factors. Among the main factors that can affect the gut microbiota during pregnancy are antibiotic administration, diet, infection, stress, and genetics (35, 36). During birth, the maternal microbiota will be vertically transmitted to the newborn, and the mode of birth affects its colonization. The composition of the microbiome acquired in early childhood is critical for shaping the immune function and metabolic state of infants and adults (37).

The early microbiome is recognized for its major influence on long-term human health and development, and variation in gut microbiome composition and function is influenced by several factors including diet (35). In recent years, researchers have become increasingly interested in the impact of dietary interventions during pregnancy on the development of the infant’s gut microbiome. Dietary elements analyzed in the mother included usual eating habits and nutritional elements (38). After adjusting for variables such as demographic characteristics, type of birth, and breastfeeding status, no independent significant impact on the infant microbiome was observed (39). It has also been shown that the administration of probiotics to pregnant and lactating mothers has an impact on the newborn, influencing its intestinal microbiota (38). Also, (232) showed how the gut microbiota can regulate neuroinflammation and motor deficits, further illustrating the potential of dietary interventions to mitigate the risks associated with MIA. The interaction between maternal diet, gut microbiota, and immune activation may be critical in determining the neurodevelopmental trajectory of the fetus. Epidemiologic evidence suggests that exposure to poor diet in early pregnancy almost doubles the risk of schizophrenia in offspring. For this reason, the diet of pregnant women, especially in the early stages of pregnancy, has an impact on epigenetic changes in the fetus (29). Dietary factors can impact gene expression through epigenetic modifications via complex mechanisms (40). Bioactive dietary components affect gene expression by altering chromatin structure (DNA methylation, histone modification), non-coding RNAs, and the activation of transcription factors (41). Nutrients and bioactive compounds found in foods influence these epigenetic processes either by directly inhibiting DNA methylation and histone-modifying enzymes or by changing the availability of substrates required for these enzymatic reactions. For instance, diets rich in polyphenols, such as catechols, can reduce enzyme activity and reactivate genes that are otherwise epigenetically silenced. Additionally, various nutrients, including folate, riboflavin, methionine, and pyridoxine, play crucial roles in one-carbon metabolism and directly affect S-adenosyl-L-methionine levels (40).

Proper prenatal nutrition is crucial for optimal brain development. Research in epidemiology increasingly shows that exposure to nutritional deprivation may increase the risk of schizophrenia (42). Nutritional deficiencies can have a significant impact on the production and functioning of neurotransmitters. Foods are recognized for their influence on mood, mainly by providing essential precursors for neurotransmitters. In addition, nutritional supplements, such as iron and folic acid, may be helpful in ameliorating symptoms of anxiety and depression (43). The most common nutritional deficiencies found in people with mental disorders include omega-3 fatty acids, B vitamins, minerals, and amino acids that are precursors to neurotransmitters (44). Adequate consumption of essential nutrients, which include protein, vitamins B and C, as well as certain trace elements, is crucial for maintaining a healthy brain and maintaining balanced levels of neurotransmitters (43). The latest update of the neurodevelopmental theory of schizophrenia has indicated that several risk factors that may act together on a genetically predisposed individual at different developmental stages, contributing to the clinical syndrome of schizophrenia. An important risk factor is vitamin D deficiency, especially during pregnancy and childhood (45).

It has been found that both prenatal and postnatal exposure to different nutritional elements or their deficiencies can lead to changes in the brain, which can predispose to neurodevelopmental disorders. Maternal nutrition can modulate gene expression through epigenetic mechanisms, methyl-donor micronutrients of one-carbon metabolism (folic acid, choline, and vitamins B6 and B12) are essential in many physiological pathways and processes, including DNA methylation (46). Maternal diet before pregnancy allows optimizing nutritional status to maintain a healthy pregnancy and fetal development. Moreover, during the period of conception, maternal nutrition is important for gamete function and health and for placental development (46).

The fetal brain consumes approximately 75% of fetal energy, suggesting that the developing brain is susceptible to nutrient restriction. Thus, it is suggested that an insufficient supply of nutrients causes different consequences depending on the stage of pregnancy, at first causing changes in neuronal proliferation, and in the earlier stage causing changes in neuronal differentiation (46). On the other hand, a high-calorie maternal diet causes reprogramming of myeloid progenitor cells, increasing the immune response throughout life (47).

Furthermore, prenatal malnutrition can disrupt myelin formation, conferring susceptibility to the pathology of schizophrenia (48). Numerous nutrients including Cu, folic acid, and choline demonstrate an important role in brain development. Preterm infants continue to accumulate macronutrient deficiencies and undergo initial growth reactions during the first weeks of hospitalization (49). There is also evidence that some micronutrient deficiencies, including low homocysteine, and vitamin D, increase the incidence of schizophrenia (50). The effects of long-chain polyunsaturated fatty acid (PUFA) supplementation on child development during gestation, infancy, and early childhood were studied, finding no significant benefit on cognition and attention. Iron deficiency is the most common deficiency nutritional deficit in the world and one of the first four cases of loss of developmental potential among children (49).Vitamin D is recognized for its important role in cell differentiation and proliferation, as well as neurotrophic actions in the brain. This micronutrient could modulate synaptic plasticity and neurotransmission, thus contributing to the optimal functioning of the nervous system (51). In addition, vitamin D plays an essential role in numerous brain processes, including neurodevelopment and neurotransmitter expression, and is valued for its neuroprotective effects (52). These neuroprotective effects are attributed to vitamin D’s ability to influence the production and release of neurotrophins, support neurotransmitter synthesis, maintain intracellular calcium homeostasis, and prevent oxidative damage to nervous tissue (53). Vitamin D has also been shown to have a significant antioxidant effect on neurons and the brain by increasing the levels of powerful antioxidant molecules such as cytochrome c and glutathione (54). This vitamin reduces oxidative stress and inflammation caused by free radicals and reactive oxygen species (ROS) by stimulating specific neurotrophic factors (55). Furthermore, it protects the brain against damage caused by ROS by increasing the levels of antioxidant molecules such as glutathione in non-neuronal cells (56).

Vitamin D deficiency is extremely common in people suffering from schizophrenia and other mental health problems. Epidemiologic research has shown that people born in late winter/early spring, in high-latitude areas, and urban settings are at increased risk of schizophrenia, indicating that this risk may be influenced by vitamin D deficiency. This association is suggested by studies of African and Caribbean migrant populations, among whom vitamin D levels tend to be low. Migrant populations from Africa and the Caribbean have increased rates of psychosis (57). Furthermore, the offspring of individuals migrating from more equatorial regions to colder climates are also at increased risk of schizophrenia, as are the offspring of city dwellers compared with those living in rural regions (58). Serum vitamin D levels were negatively related to the severity of symptoms of psychosis. The lower the vitamin D concentrations, the higher the overall negative symptom scores 124 (59). A minor way to obtain vitamin D is through diet, as some foods contain this vitamin such as fatty fish. Vitamin D can also be consumed by taking supplements or eating fortified foods such dairy products. From a global perspective, vitamin D deficiency is among the most common micronutrient deficiencies (60).

As a fat-soluble vitamin, Vitamin D has diverse biological effects on the body (61). Vitamin D deficiency is linked to a wide variety of neuropsychiatric problems and neurodegenerative disorders (62). The effects of vitamin D in the brain are diverse, including promoting antioxidant and neurotrophic action, as well as regulating various neurotransmitter systems (including dopamine). In animal studies, the offspring of vitamin D-deficient rodents show brain structural changes similar to those seen in schizophrenia, such as thinning of the cortex, enlargement of lateral ventricles, and increased brain size due to increased proliferation and reduced shedding of neurons (63). In psychotic disorders, vitamin D has been positively associated with peripheral gray matter volume, possibly indicating a neuroprotective effect (64).

Some time ago, it was noted that schizophrenia may be associated with vitamin B deficiency (65). B vitamins are crucial in cellular metabolism functions, including homocysteine regulation, transmission processes, and oxidation–reduction reactions. Experts have emphasized that certain B vitamins, such as vitamin B6 (pyridoxine), vitamin B12 (cobalamin), folic acid, and choline, may be linked to the onset and progression of schizophrenia (66, 67). Studies have determined that severe folic acid deficiency increases the likelihood of neurodevelopmental disorders, psychiatric disorders, and dementia, while changes in vitamin B12 levels can lead to neuropsychiatric problems, mental disorders, reduced cognitive abilities, mood disorders, aggressive behavior, fatigue, and paranoid psychosis (68).

Neurotropic B vitamins, such as B1 (thiamine), B2 (riboflavin), B3 (niacin), B6, B9 (folate), and B12, are important for the health of the central nervous system, playing essential roles as coenzymes, among other functions. Mammals cannot produce B vitamins, so they must obtain them from their food in sufficient quantities. Most B vitamins come from plant or animal sources, such as meat, eggs, and dairy. Only vitamin B12 comes from an animal source. A lack of B vitamins has been linked to various neurodevelopmental issues, with one of the most significant being schizophrenia (69). Many studies have found that the main etiological factor for schizophrenia is a deficiency in vitamin B12 and elevated homocysteinemia. Not only can a deficiency in vitamin B12 cause schizophrenia, but vitamins B6 and B9 can also contribute to the condition, and symptoms can be reduced by supplementing these vitamins alongside antipsychotics (70).

Vitamin D deficiency is one of the most studied risk factors for schizophrenia, being associated with cognitive impairment, including difficulty with attention and memory. In the context of schizophrenia, low vitamin D levels are correlated with increased symptom severity, including more pronounced negative symptoms and increased cognitive impairment (55). Low vitamin D, either at birth or in the postnatal periods, may contribute to the development of brain developmental disorders, including schizophrenia (71). Prenatal exposure to nutritional deficiencies, such as vitamin D deficiency, has been identified as a determinant of schizophrenia (72). Regarding vitamin C, its deficiency could worsen the symptoms of schizophrenia (73). Vitamin B9 plays a crucial role in fetal brain development, and its deficiencies have been associated with increased risks of schizophrenia (74).

Maternal iron deficiency is also considered a risk factor for schizophrenia, and future research may help reduce the risks for these disorders (75, 76). Maternal protein deficiency and prenatal exposure to starvation are considered significant factors in the risk of schizophrenia (77). Furthermore, glycine deficiency leads to behavioral changes responsible for the cognitive and negative symptoms of schizophrenia (78). Also, prolonged dietary fiber deficiency can lead to changes in the composition of the gut microbiome, influencing the development and progression of various schizophrenias (79). Epidemiologic, biochemical, and genetic association studies have demonstrated that folate deficiency is a risk factor for schizophrenia (80). Essential for the synthesis of DNA and neurotransmitters, it plays an important role in regulating gene expression. An adequate amount of folate during pregnancy can reduce the risk of congenital anomalies, such as neural tube defects. Research has shown that a lack of folate during pregnancy can significantly increase the likelihood of schizophrenia in offspring. Previous studies have linked low blood levels of folic acid to more severe symptoms among patients with schizophrenia (81).

There are natural amounts of folate in various foods such as green leafy vegetables, fruits, nuts, beans, seafood, eggs, dairy products, meat, poultry, and cereals. Spinach, liver, asparagus, and Brussels sprouts are among the foods rich in folic acid (82) which is essential for the proper functioning of neurons. It is confirmed to be crucial for people with schizophrenia in managing energy levels. A lack of folic acid, either alone or in conjunction with monoamine precursors such as vitamins B6 and B12, can increase the risk of schizophrenia or exacerbate existing mood disorders (70). As a complex, B vitamins are essential for various brain functions. Further studies conducted to investigate the precise mechanisms of B vitamins in brain functions will provide the possibility of using B vitamins to improve mental health and prevent psychological disorders (83). There is a known relationship between the amount of alpha-linolenic acid in the maternal diet during gestation and lactation and the accumulation of these compounds in the fetus’s or newborn’s brain until an optimal level is reached (84).

Similar to other major neuropsychiatric illnesses, schizophrenia usually begins in the late teens or early 0 s, with a slightly delayed onset in women (85). Disruption of normal brain growth during prenatal or early postnatal periods can lead to brain dysfunction, suggesting that an affected central nervous system may play a crucial role in the development of this condition. Indeed, individuals with schizophrenia, compared to healthy individuals, show a significant reduction in total brain volumes and density of gray and white matter, while showing significant increases in the volumes of the lateral and third ventricles (86, 87).

Schizophrenia is viewed as a brain dysfunction because the control of all functions and behaviors is executed by the human brain. The brain contains the highest amount of lipids of all the body’s organs (86). Lipids and lipid intermediates are indispensable elements in the organization and functioning of the brain. Brain tissue contains the second-highest percentage of fat after adipose tissue, and fat makes up half of the brain’s dry weight (88). Over 60% of the lipids present in the membrane are phospholipids. Brain phospholipids include two categories of polyunsaturated fatty acids: omega-3 and omega-6. Docosahexaenoic acid (DHA) is crucial for the normal development of the nervous system and is particularly important in maintaining biological functions (receptor binding, neurotransmission, signal transduction, and cognitive abilities, including learning and memory) (86).

Humans cannot naturally produce omega-3 and omega-6 polyunsaturated fatty acids and must obtain these essential substances from their diet. This essential nature could support the substantial epidemiological evidence showing an inverse relationship between omega-3 fatty acid intake and the prevalence of psychiatric disorders (89). Fatty fish is a food rich in essential fatty acids and vitamin D, both of which may be implicated in the development of schizophrenia. It is suggested that a low maternal intake of fish and seafood during pregnancy may increase the risk of low IQ and suboptimal neurological development in the child, thereby linking to a higher risk of mental disorders in adulthood, such as schizophrenia (90). The importance of omega-3 polyunsaturated fatty acids in the maintenance of physical health is well known, as they are involved in a variety of physiological functions related to neurogenesis, neurotransmission, and neuroinflammation, and play essential roles in brain development, activity, and aging (91).

Lack of long-chain omega-3 polyunsaturated omega-3 polyunsaturated fatty acids impairs neuronal development and may contribute to various mental health problems (92). In schizophrenia, changes in the structure of phospholipids in cell membranes may influence various aspects of pathophysiology, such as neurotransmission, immune activation, and antioxidant protection. Studies have shown a deficiency of omega-3 polyunsaturated fatty acids in patients with schizophrenia compared to healthy individuals, thus supporting this hypothesis. The addition of omega-3 polyunsaturated fatty acids could be useful in preventing the onset of psychosis, especially among those at increased risk (93).

Various experimental studies have clearly shown the influence of omega-3 fatty acids on brain structures and activities. For proper differentiation and function, dissociated brain cell cultures require omega-3 and omega-6 fatty acids (94). Simultaneously, a lack of alpha-linolenic acid in the diet affects brain development, disrupting the structure and chemical composition of neuron membranes (including oligodendrocytes and astrocytes), myelin, and nerve endings (95). This is accompanied by the appearance of neurosensory and behavioral disorders. Omega-3 fatty acids are considered important for their potential to increase favorable side effects and their ability to repair metabolic issues of phospholipids in synaptic membranes, which can lead to dysfunction of dopaminergic and serotonergic neurotransmitter receptors. Omega-3 fatty acids can mitigate the negative consequences of medications through their anti-inflammatory actions and pose a low risk of causing damage. Supplementation and diet are two ways to increase omega-3 intake. Equally crucial are dietary changes to reduce the amount of omega-6 consumed. For example, industrially derived seed oils, such as safflower, sunflower, and vegetable oils, contain large amounts of omega-6 fatty acids (96).

Pregnancy is characterized by the rapid growth of maternal and fetal tissues, which involves an increased demand for energy and nutrients (97). Lack of essential nutrients during critical growth phases can cause long-term brain dysfunction (98). The food consumed by the mother during pregnancy, as part of the period known as the “first 1,000 days,” is considered extremely important for the optimal development of offspring (97), reducing the risk of diseases throughout life, and overall long-term health. Currently, many patients with schizophrenia continue to experience residual symptoms and cognitive difficulties despite optimal pharmacological treatment. Although there is a continual search to enhance the effectiveness of antipsychotic agents, no strategy has proven to be markedly superior.

## 4 Obesity in schizophrenia: the role of diet and antipsychotics

**Figure 4:**
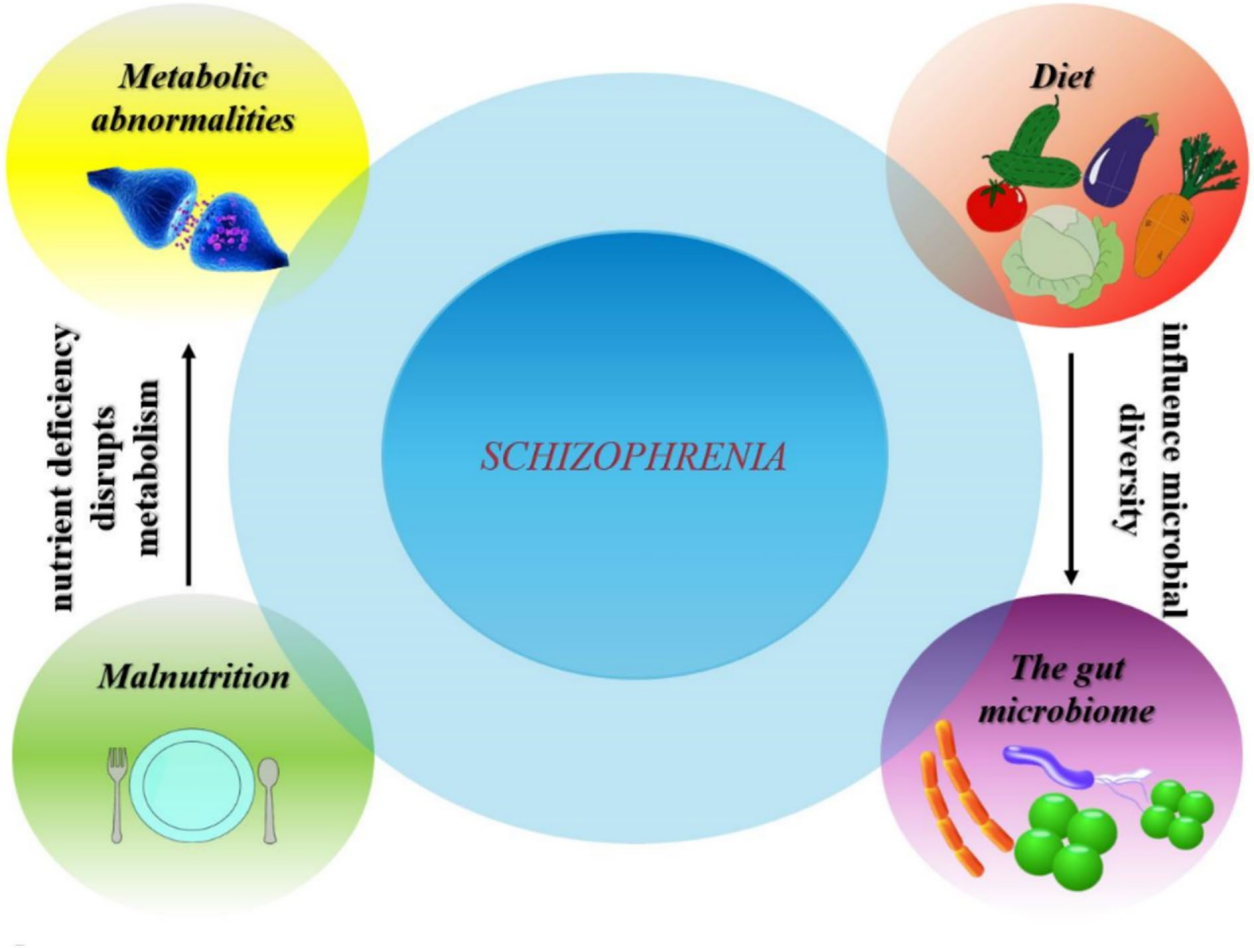
Complex interactions between diet, gut microbiome, metabolic abnormalities and malnutrition in relation to schizophrenia prevalence.

Numerous factors contribute to weight gain in patients suffering from schizophrenia or psychosis (Figure 4). The main contributing factors are considered to be a sedentary lifestyle, unhealthy eating habits, genetic susceptibility, and antipsychotic treatment. People with severe mental disorders are at increased risk of obesity, cardiometabolic risk factors, and associated morbidity and mortality compared to the general population (99, 100). Weight gain caused by antipsychotics is a significant issue in the care of patients with psychosis (101). In addition to causing weight gain, antipsychotics are also known for their effect on glucose metabolism, increasing cholesterol and triglyceride levels, and inducing high blood pressure, leading to MetS (102). The prevalence of MetS is high among individuals with schizophrenia. The frequent occurrence of both MetS and obesity in this population leads to a reduced life expectancy and an increased mortality rate (101). Changes in body structure are observed early in individuals experiencing their first episode of psychosis, with a greater predisposition to being overweight and obese. Weight gain occurs rapidly after starting antipsychotic treatment and continues, albeit at a slower rate, over the following years (103).

Metabolic abnormalities already appear at the onset of psychosis, before the start of antipsychotic treatment (104). Misiak et al. (104) confirm that patients with schizophrenia spectrum disorders show a few metabolic abnormalities related to impaired glucose metabolism, high triglyceride levels, low HDL levels, high levels of visceral fat deposition, tolerance to impaired glucose, insulin resistance and type 2 diabetes (105). Individuals with schizophrenia are at a significantly higher risk of developing the aforementioned metabolic disorders (106). Many antipsychotics are associated with weight gain, lipid disturbances, and glucose dysregulation, which contribute to the development of (MetS) (107). MetS is much more prevalent in people with schizophrenia, presenting a significant cardiovascular risk and mortality (108). Although changes in the metabolic profile have been reported in drug-naïve patients, it has been suggested that metabolic dysregulation in schizophrenia may be a consequence of common genetic bases for psychotic and metabolic disorders. This hypothesis has been proven by some studies showing that genetic polymorphisms in TCF7L2, AKT1, and TSPAN8 genes could increase the risk of diabetes and schizophrenia (109). Metabolic dysfunction has not only been reported in schizophrenia, but also Alzheimer’s disease, major depressive disorder, and bipolar disorder. Symptoms of metabolic abnormalities ultimately result from abnormal brain function (105).

Insulin resistance is another critical aspect of metabolic health that warrants attention in this context. Research indicates that insulin resistance is significantly elevated among patients with schizophrenia, often exacerbated using antipsychotic medications. This condition not only contributes to the risk of developing type 2 diabetes but also negatively impacts overall brain function (110). Interventions aimed at improving insulin sensitivity through dietary modifications have shown promise in enhancing cognitive performance and metabolic health in these patients (111). Given the intertwined nature of obesity, insulin resistance, and antipsychotic treatment, addressing these metabolic disturbances through comprehensive lifestyle changes could be vital for improving both physical and mental health outcomes in individuals with schizophrenia.

Several commonly prescribed medications can cause weight gain and, in the case of prolonged treatment, can lead to clinically significant obesity (112). These negative consequences of medication are key factors leading to decreased quality of life and premature death due to cardiovascular disorders in patients with severe mental disorders compared to the rest of the population (113). Explanations given for drug-induced weight gain include increased appetite, changes in food preferences toward carbohydrates, and alterations in nutrient metabolism. Other significant factors can explain medication-induced weight gain, such as impaired metabolism due to decreased thyroid function, fluid retention, and the anabolic effect of steroids (112).

The treatment of schizophrenia has evolved considerably over time, reflecting important advances in the understanding of the pathophysiology of the disease and the development of therapeutic strategies. One of the earliest treatments for schizophrenia, called insulin shock therapy, was introduced by the Austrian doctor Manfred Sakel in 1933. This treatment involved the administration of large doses of insulin to induce a hypoglycemic coma in patients, who were then brought out of the coma by glucose injections (114). Dysregulation of insulin action has been associated with the pathophysiology of schizophrenia and type 2 diabetes (115, 116). Insulin plays a crucial role in regulating dopamine levels in the striatum, controlling feeding behavior, and maintaining peripheral glucose homeostasis (115). Weight gain caused by insulin therapy can be a major challenge for patients with type 2 diabetes who are already overweight (117). In 1938, electroconvulsive therapy was introduced as an effective treatment for schizophrenia and other psychotic disorders. However, with the advent of chlorpromazine in the 1950s and the subsequent development of new pharmacological agents, the use of this therapy has declined considerably (118). The introduction of antipsychotics in the 1950s revolutionized the treatment of schizophrenia, providing a particularly effective therapy for managing the positive symptoms of the disease (119). In 1950, chlorpromazine was introduced into clinical investigations in France. The use of this drug has highlighted the variability of treatment response in schizophrenia (120). The evolution of schizophrenia treatments has demonstrated significant adaptation in therapeutic approaches, reflecting a deeper understanding of the pathophysiology of schizophrenia and the impact of side effects, such as weight gain, on patients.

Antipsychotic drugs are considered a vital therapeutic choice for many patients with schizophrenia and other psychotic disorders. To offer maximum benefits, the medication must have acceptable side effects and be taken according to medical guidelines (112). A common negative consequence of many antipsychotics is weight gain. The way weight increases seems to differ depending on the medication, which may be caused by variations in the action of the medications on different neurotransmitter systems such as serotonin, dopamine, choline, histamine, and others (121).

Secondly, antipsychotics are prescribed as the main treatment for alleviating symptoms, preventing the relapse of schizophrenia, and regulating dopamine levels to normal (59). Despite these aspects, the administration of antipsychotic medications can frequently have side effects such as increased appetite and body weight by disrupting ghrelin and leptin, thus altering food intake and causing weight gain in patients with schizophrenia (122).

The addition of atypical antipsychotics to the field of psychopharmacology has represented a significant advance in the therapy of schizophrenia, providing an effective way to treat the positive and negative symptoms of psychosis. Before their widespread use, there was concern about treatment-related weight gain and the apparent increased risk of diabetes mellitus (123). Among people with schizophrenia, the prevalence of obesity is double that of the general population, affecting more than half of this patient category. In addition to the negative psychosocial impact (distorted self-image and societal stigma) and non-compliance with medication treatment, people with schizophrenia appear to be extremely sensitive to the harmful medical consequences of obesity, such as “metabolic syndrome,” which includes a set of cardiovascular risk factors such as abdominal adiposity, insulin resistance, impaired glucose tolerance, dyslipidemia, and high blood pressure (26). Moreover, antipsychotics differ significantly in their potential to cause metabolic disturbances. Clozapine and olanzapine present the highest risks, whereas ziprasidone and aripiprazole are associated with minimal metabolic risks (124).

**Figure 5:**
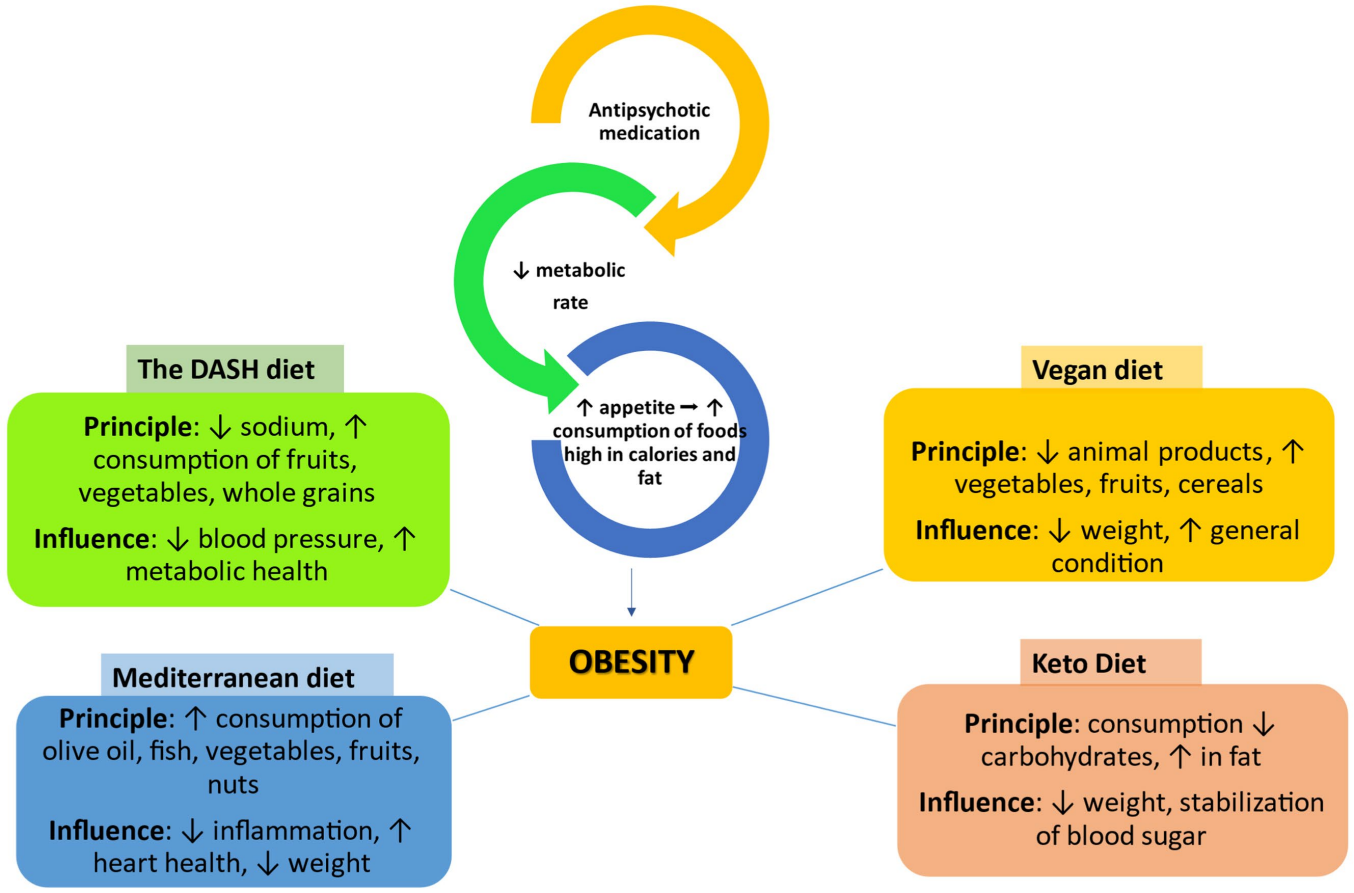
The impact of different types of diets on obesity caused by antipsychotic drugs ↑, increase/improve; ↓, decrease; →, leads to, DASH, dietary approaches to stop hypertension.

Antipsychotics can lead to increased appetite (Figure 5). People suffering from schizophrenia make inadequate food choices, which means that any increase in appetite is likely to lead to increased consumption of snacks and convenient foods that are high in fat and sugar (125). Patients diagnosed with schizophrenia have an increased appetite at meals, eat more quickly, and have more meals per day compared to the general population. This irregular food consumption eventually leads to excess energy intake, which contributes to the onset of obesity. Skipping breakfast and irregular eating can lead to compulsive eating, which can contribute to obesity (126). People diagnosed with schizophrenia adopt inadequate eating habits, characterized by increased consumption of saturated fats, sugar, and alcohol while having reduced intake of fish, vegetables, and fruits, which could affect cognitive function (59).

However, in recent years, current treatments have been shown to have a significant impact on oxidative pathways and can reverse pro-oxidative states in schizophrenia (127). Oxidative stress may represent a primary driver of disease and toxicity, and once damage begins, oxidant therapy cannot inhibit the progression of tissue damage due to other factors becoming dominant in the pathology (128). To defend against oxidative damage, organisms have developed defenses dependent on antioxidant enzymes and damage repair. In response to oxidants, the ability to detoxify oxidants and repair oxidative damage is enhanced. Improving these defenses is the main strategy underlying antioxidant therapy (128). Research on the therapeutic use of antioxidants in schizophrenia can be grouped into two main groups, namely for psychopathology and secondary effects (129). It has been found that fruits and vegetables rich in antioxidants included in the daily diet strengthen the ability to fight against infections and diseases. By investigating antioxidant modulation as a therapeutic target for the treatment of schizophrenia, mechanisms of action were found to vary between compounds. For example, Omega-3, exhibits direct antioxidant properties, acting by protecting against oxidative attack and by strengthening lipid membranes (127). N-acetylcysteine may provide tangible benefits for the clinical syndrome of schizophrenia by acting predominantly on the glutathione pathway, and vitamin E may provide beneficial effects on the glycemic effects of antipsychotics (127, 129). Magalhães et al. (127) state that *Ginkgo biloba* has a positive effect on psychotic symptoms in the short term, unlike other antioxidants such as allopurinol and selegiline that showed no real differences compared to placebo.

Increased food intake associated with obesity has been observed in the early stages of therapy with chlorpromazine and depot antipsychotic medications (a special formulation of the drug, which is administered by injection and is slowly released into the body over several weeks) (125), and the problem of weight gain and diabetes mellitus has been highlighted in connection with atypical antipsychotic treatment (25). The way antipsychotic medications influence food intake is probably related to their impact on dopamine and serotonin receptors (25). After treatment with conventional and atypical antipsychotic agents, high levels of circulating leptin have been observed. Leptin, a hormone fat cells produce, influences the hypothalamus to suppress hunger. The body of obese individuals is often resistant to this characteristic of leptin (130, 131).

Differences in weight gain tendency vary between medications, with the greatest effects observed with olanzapine and cloza (103). The increased risk of weight gain with these medications is due to their action on serotonin, dopamine, histamine, and muscarinic receptors (101). Although many studies have focused on new-generation antipsychotics, weight gain can also occur with older antipsychotics, with chlorpromazine being linked to the most significant weight gain. Antipsychotics influence energy levels and change the composition of the gut microbiome, which could lead to weight gain. Appetite control is complex and involves multiple neuropeptides, some of which are influenced by antipsychotic medications (103).

Early dietary interventions in schizophrenia included suggestions about the possible effectiveness of gluten-free diets in reducing symptoms or disease severity (132). Dietary interventions may help prevent schizophrenia or delay the onset of symptoms. These balanced diets include nutritious plant-based foods and high-quality protein and are associated with a reduced risk of schizophrenia and improved symptoms. Furthermore, a diet high in saturated fat and sugars is linked to a more severe course of schizophrenia (133). People with schizophrenia have been found to eat a poor diet, have high rates of smoking, and exercise little. Although nutritional factors play a key role in the development of diabetes in patients with schizophrenia, they can affect both the outcome and severity of schizophrenia. In turn, diet is determined by other factors such as social, political, and cultural.

People with schizophrenia consume less dietary fiber, antioxidant vitamins, and fruits and vegetables in extremely low proportions. They are consuming a diet known to promote MetS, i.e., high in saturated fat and low in fiber, but with a high glycemic load (25). In addition to antipsychotic drugs, there are other proposed ways to control refractory symptoms of schizophrenia. A high-fiber diet, the DASH diet, and the Mediterranean diet may become beneficial in reducing metabolic, cardiovascular, and immune parameters related to premature mortality in schizophrenia (134).

The ketogenic diet (KD, also known as metabolic therapy) consists of a high-fat, low-carbohydrate diet (135, 136). Ketogenic diets are high-fat, low-carbohydrate diets (137). This provides an alternative source of energy for the brain, replacing glucose, and normalizes schizophrenia-like behaviors (138). In addition, it has been observed to have a very good therapeutic role in patients with intractable seizures (137). It is noteworthy that the KD normalizes pathological behaviors in an animal model of schizophrenia (139, 140). Sethi et al. (136) confirmed that participants with schizophrenia showed an average improvement of 32% according to the Brief Psychiatric Rating Scale. Furthermore, in another study, a reduction in long-term schizophrenic symptoms was observed after initiation of a low-carbohydrate ketogenic diet used for weight loss (141). However, KD has some common side effects, including headache, fatigue, and constipation (136). This dietary intervention mimics the effects of fasting and has a role in reducing glutamate toxicity, promoting the inhibitory tone of GABA, and reducing the formation of ROS, thus all of these contribute to the improvement of neuronal function (142).

The Mediterranean diet is one of the healthiest diets characterized by well-balanced proportions of fatty acids and a high consumption of fruits, vegetables, nuts, and olive oil (143). This diet has been suggested to patients with schizophrenia because this disease occurs along with other comorbidities such as obesity, (MetS), and cardiovascular disease (144). The Mediterranean diet is inspired by the eating habits of Mediterranean countries and the Middle East (145), emphasizing fresh vegetables, grains, olive oil, and herbs; limiting meat consumption, is known to reduce blood cholesterol (146). In addition, it has been noticed that it has positive effects on diabetes mellitus, cardiovascular diseases, arthritis, and cancer (147, 148). Purslane is notable for having the highest levels of omega-3 fatty acids of any land plant, which are essential for heart health and may help prevent strokes and heart disease. It is also rich in vitamins A and C, as well as beta-carotene, which contribute to eye health and immune function. The antioxidants present in purslane help fight oxidative stress and may reduce the risk of cancer (149).

In addition, purslane is a good source of essential minerals such as magnesium and calcium, which are important for bone health and metabolic functions. For example, a detailed analysis of the mineral composition of purslane revealed its significant levels of magnesium (68 mg/100 g) and calcium (65 mg/100 g), among other essential minerals (150).

Research suggests that purslane may also support diabetes management by lowering blood sugar levels and improving lipid profiles. Its anti-inflammatory properties further enhance its potential as a therapeutic food for chronic conditions associated with inflammation (146). In short, incorporating purslane into the Mediterranean diet not only aligns with its principles of consuming nutrient-dense foods but also provides additional health benefits that can help prevent and manage various chronic diseases.

The Dietary Approaches to Stop Hypertension (DASH) diet is a diet designed to lower blood pressure. DASH is a low-sodium, low-fat diet designed to treat high blood pressure (151, 152). As with ketogenic diets, DASH diets are effective for weight loss (153). In a DASH dietary pattern, special emphasis is placed on low-fat milk and dairy products, whole grains, lean meats, fruits, and vegetables, along with reducing sodium intake. However, studies of the DASH diet in schizophrenia are insufficient and limited (154). DASH could be broken down by a high intake of fruits, vegetables, grains, poultry, fish, and a low intake of saturated fat, red meat, and sugary drinks (59).

Moreover, mineral and vitamin supplementation has been found to reduce psychiatric symptoms in people with schizophrenia (155). It has been found that supplementation with B vitamins may help reduce psychopathological symptoms in this population. Omega-3 fatty acids were more specifically associated with decreased positive symptoms and general psychopathology. However, the relationship between diet and psychosis could be bidirectional (Table 1).

**Table 1:** Nutritional deficiencies relevant to mental health: micronutrients and macronutrients related to schizophrenia.

Category	Nutrient	Possible association with schizophrenia	References
Micronutrients	Vitamin D	Low levels of vitamin D, either at birth or during postnatal periods, have been indirectly implicated in various neurodevelopmental disorders.	(71)
		Low maternal vitamin D can affect neural development and lead to various mental illnesses, including SZ.	(188)
		It is essential for fetal brain development and may play an important role in the early stages of psychotic spectrum pathogenesis.	(74)
		Maternal vitamin D deficiency in early pregnancy is linked to an increased risk of SZ in offspring.	(189)
		*In utero* exposure to nutritional deficiency is a determinant of SZ.	(72)
	Vitamin D3	D3 deficiency is linked to a higher risk of developing SZ in adulthood.	(3)
	Vitamin B9	It is vital for fetal brain development and may represent the initial steps in the pathogenesis of psychotic disorders.	(74)
		Implicated in the association between starvation and SZ.	(77)
	Vitamin C	Vitamin C has been suggested to play a role in exacerbating the symptoms of SZ.	(73)
Minerals	Iron	Maternal iron deficiency in the third trimester may be a risk factor for SZ spectrum disorders in offspring.	(75, 76)
Macronutrients	Proteins	Maternal protein deficiency and prenatal famine exposure may contribute to the increased risk of SZ through the link between low birth weight and this risk.	(77)
	Essential amino acids	Serine racemase deficiency or D-amino oxidase overactivity, which degrades D-serine, is associated with SZ.	(190)
		Glycine binding deficiency, observed in experimental mice, leads to behavioral changes responsible for cognitive and negative symptoms of SZ.	(78)
	Omega-3 fatty acids	n-3 PUFA deficiency in the blood or erythrocyte membranes of SZ patients.	(191)
	Fiber	Prolonged dietary fiber deficiency can cause lasting changes in the gut microbiome, impacting the development and progression of various diseases.	(79)

SZ – schizophrenia.

The introduction could benefit from a more in-depth discussion of the biological mechanisms through which diet influences schizophrenia beyond just oxidative stress and inflammation (e.g., epigenetic changes, neuroplasticity) (143). Co-supplementation with vitamins C, E, and omega-3 fatty acids decreased several psychiatric scores (59). Supplementation with omega-3 fatty acids might improve disease progression, although conflicting data also exist (86). There is increasing evidence to suggest that vitamin supplementation, particularly folic acid, vitamin B12, and vitamin D, may be important in the treatment of certain subgroups of patients (156). A study of supplementation with vitamins E and C (E/C, 400 IU:500 mg) and omega-3 PUFA fatty acids (eicosapentaenoic acid/ docosahexaenoic acid, 180/120 mg) in a group of 33 patients with schizophrenia showed a reduction significant of psychopathological symptoms (157). Valipour et al. (158) confirm that vitamin D supplementation has not yet been addressed in improving the symptoms of schizophrenic patients with vitamin D deficiency and their response to antipsychotic drugs.

The DASH diet and the Mediterranean diet are lifestyle changes that have positive effects on the prevention, risk reduction, and management of chronic disease. Various studies have shown that strict adherence to these dietary choices can have beneficial effects on schizophrenia (3). People suffering from schizophrenia need to be carefully initially evaluated and constantly monitored for physical health parameters. Plans to treat and prevent should incorporate promoting a healthy lifestyle, proper nutrition, and adequate levels of physical activity (159). In this context, it is essential to understand how various types of diets can influence antipsychotic-induced obesity (Table 2), a common problem in the treatment of schizophrenia.

**Table 2:** Effects of different types of diets on antipsychotic-induced obesity in patients with schizophrenia.

The type of diet	Description	Effects on obesity	Mechanism of action	References
Ketogenic diet	High in fat, moderate in protein, low in carbohydrates	Weight loss, reductions in visceral adipose tissue, glycosylated hemoglobin and triglycerides	Mood stabilization and cognitive function	(192)
Mediterranean diet	Rich in vegetables, fruits, fish, olive oil, whole grains	Reduces the risk of obesity and improving metabolism	Improves metabolic and immunity outcomes	(139, 193)
Diet DASH	Rich in vegetables, fruits, low-fat dairy products, whole grains	lowers triglycerides, lowers fasting blood glucose and improves insulin resistance	Reduce inflammation and oxidative stress, improve vascular and metabolic function.	(154, 194)
Calorie restriction diet	Caloric restriction with balanced intake of macronutrients	Improves weight and metabolic markers	Reduce inflammation and oxidative stress, improve insulin sensitivity and metabolic health.	(195) (196, 197)
High protein diet	Includes animal and vegetable protein sources	Maintain muscle mass, reducing body fat	Stabilizing blood sugar, reducing inflammation and improving neural function.	(198, 199)
Gluten-free diet	Exclude all foods containing gluten	Improvements in negative symptoms	Reducing inflammatory responses	(132)

DASH, dietary approaches to stop hypertension.

Excess weight, which represents a major risk for (MetS), cardiovascular diseases, and premature mortality, is a common physical health problem among patients with schizophrenia spectrum disorder (160). Since eating habits influence the quantity and type of food consumed, they can prevent or control obesity, making them modifiable factors. It has been observed that a regular or healthy eating pattern impacts overall and abdominal obesity in patients with schizophrenia, and these effects differ by gender (126).

## 5 Impact of diet on oxidative stress and inflammation in schizophrenia

Free radicals are naturally formed under normal physiological conditions and have a significant impact on various biological processes (161). However, when these molecules are produced in greater quantities than necessary, they can trigger spontaneous chain reactions that may lead to consequences such as abnormal neuronal development and impaired neuronal function. Free radicals are considered unstable due to the presence of one or more unpaired electrons, which makes them extremely reactive (126, 162).

Oxidative stress is a complex biological process defined by the excessive generation of ROS, which disturbs the body’s redox balance and leads to oxidative damage (163). This occurs when there is a disturbance in free radicals, as well as reactive oxygen and nitrogen species, derived from both normal metabolic processes, including neurotransmitters associated with schizophrenia, such as dopamine and glutamate, and various environmental exposures. Increased oxidative stress in schizophrenia is influenced by several interconnected factors, which include likely contributions from the environment, genetic predispositions, and immune system dysfunctions (164). The insufficient effectiveness of the antioxidant system in countering free radical production can lead to cellular membrane damage, resulting in dysfunctions that may affect neurotransmission and contribute to the symptoms of schizophrenia. This is closely linked to increased oxidative stress, which ultimately leads to various pathological outcomes, including lipid peroxidation (165). Radicals can interact with polyunsaturated fatty acids, and the cell membranes of tissues exposed to high oxygen levels, such as the brain, are at risk of oxidizing their unsaturated fatty acid content in their phospholipids (162). The human body’s defense system includes antioxidant enzymes such as superoxide dismutase, glutathione peroxidase, and catalase. These enzymes stop the initiation of chain reactions of reactive species. Substances like glutathione, vitamin E, and vitamin C are non-enzymatic antioxidant components that bind to reactive species and stop the extension of chain reactions (166). To better understand the impact of diet on oxidative stress and inflammation in schizophrenia, it is useful to analyze specific parameters that measure the levels of antioxidant enzymes and non-enzymatic antioxidant components.

A study found that patients on antipsychotic treatment showed lower scores on the Brief Psychiatric Rating Scale (BPRS) and Positive and Negative Syndrome Scale (PANSS) after taking vitamin C, vitamin E, and omega-3 fatty acids (157). However, it is unclear if the effects are solely due to vitamin supplementation because they were given in combination. In a study conducted by Bentsen et al. (167), patients with schizophrenia taking antipsychotic medication were given vitamin C (364 mg/day) and vitamin E (1,000 mg/day) in a randomized placebo-controlled trial. Results showed that the vitamin supplementation hindered recovery from acute psychosis in patients with low levels of red blood cell PUFAs compared to those who received a placebo. The writers suggest that at a certain level, vitamin E may function as a pro-oxidant in the absence of sufficient antioxidant activity, leading to increased oxidative stress; it might also block the positive effects of *γ* (gamma)- and *δ* (delta) tocopherols. They propose that vitamin C could offset some negative impacts of vitamin E (156).

Superoxide dismutase, catalase, and glutathione peroxidase make up the enzymatic antioxidant defense system in the human body, stopping the start of reactive species chain reactions (168). The inability of antioxidants to protect against free radicals can damage cell membranes, causing dysfunctions that could influence neurotransmission and ultimately symptoms in schizophrenia (165). The non-enzymatic antioxidant components are compounds such as glutathione (GSH), vitamin E, and vitamin C, which react with reactive species and thus prevent chain reactions from spreading (168).

Among patients with schizophrenia, research has shown varying results regarding SOD levels, but most of the evidence indicates an increase in activity as a compensatory response to increased oxidative stress, although low or unchanged levels are also observed in other studies, highlighting the complexity and variability of oxidative balance in schizophrenia. As with SOD, research on CAT levels in patients with schizophrenia has had varied results, reflecting the complexity of oxidative stress and antioxidant defense mechanisms in this disorder. CAT levels vary with oxidative stress, nutritional status, genetic and epigenetic factors, chronic inflammation, and drug effects. Studies suggest that MDA levels are usually frequently elevated in patients with schizophrenia, rather than reduced. Elevated MDA levels indicate an increase in oxidative stress in these patients, which is a significant factor in the pathophysiology of the disorder.

Low levels of GPx in patients with schizophrenia are due to several factors, such as chronic oxidative stress, nutritional deficiencies (especially selenium), and the chronic inflammation associated with this condition. Mitochondria are organelles involved in cellular energy production, but their function may be impaired in patients with schizophrenia (169–171). Improving mitochondrial function through dietary therapy may normalize energy metabolism and restore neuronal function. All of these can lead to reduced activity or reduced levels of GPx, which is essential for defending against free radicals and maintaining the health of the nervous system. Several studies have consistently observed lower glutathione levels in patients with schizophrenia. These low levels may be caused by a few factors, such as increased oxidative stress, deficient synthesis, nutrient deficiency, mitochondrial dysfunction, chronic inflammation, environment and lifestyle, and the effects of medications.

Deficiencies of vitamins E and C in patients with schizophrenia may be caused mainly by increased oxidative stress (Table 3), poor diet, lifestyle factors (such as smoking), metabolic changes, and potential effects of medications. All these factors together contribute to a decreased efficiency of these essential antioxidants, increasing oxidative stress and possibly affecting the pathophysiology of schizophrenia. Treating the lack of nutrition through diet, lifestyle changes, and possibly the addition of supplements could help these individuals.

**Table 3:** Biochemical changes of some parameters related to oxidative stress in patients with schizophrenia.

Parameter category	Evaluated parameter	The level found relative to the normal range	References
Antioxidant enzyme	GPx	Normal level	(200)
		Low level	(172, 201–207)
	SOD	Low level	(200, 202, 207, 208)
		Increased level	(209–216)
	CAT	Increased level	(200, 211, 212, 217)
		Low level	(204, 208)
	GSH	Low level	(218–222)
Biomarkers	MDA	Increased level	(200, 202, 204, 211, 214, 216, 223–228)
Antioxidants	Vitamin E	Low level	(214, 225, 229)
	Vitamin C	Low level	(225, 227, 230, 231)

CAT, catalase; GPx, glutathione peroxidase; GSH, glutathione; MDA, malondialdehyde; SOD, superoxide dismutase.

It is important to monitor these parameters to evaluate the effectiveness of dietary interventions in reducing oxidative stress and inflammation in patients with schizophrenia. It has been proven that high levels of ROS and dysfunction of the antioxidant system can cause significant damage to neuronal structure. Cognitive decline and abnormal behaviors have been associated with oxidative state deterioration. Thus, schizophrenia symptoms may arise due to the degradation of neuronal lipid membranes in certain areas or connections, caused by the excessive presence of ROS (172).

Advanced glycation end products (AGEs), which are known to induce oxidative stress, have garnered significant attention in the context of schizophrenia. These compounds bind to neuronal membranes and proteins, inhibiting cellular function and potentially disrupting neurotransmitter balance, which may lead to neuronal death (164, 173). Recent studies suggest that AGEs may play a dual role in the disease process. On one hand, they are implicated in neurodegeneration through their ability to generate ROS, which can further damage cellular structures and impair neuronal activity. On the other hand, AGEs may also influence the immune response by modulating glial cell function, promoting chronic inflammation that is often observed in schizophrenia (174, 175). This interplay between oxidative stress and inflammation underscores the complexity of schizophrenia’s pathophysiology and highlights the need for further research into therapeutic strategies that target these mechanisms (176).

**Figure 6:**
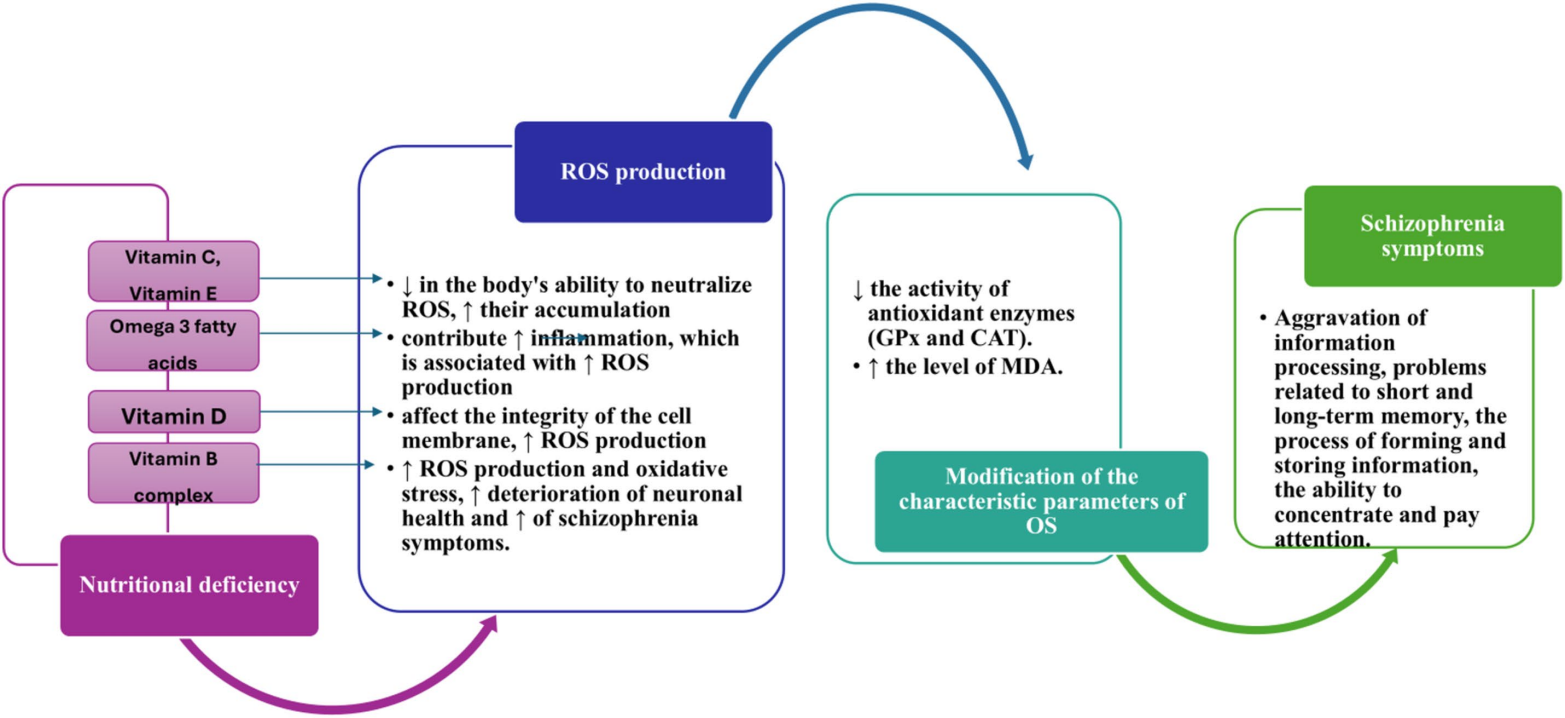
Impact of nutritional deficits on ROS production and exacerbation of schizophrenia symptoms ↑, increase; ↓, decrease; CAT, Catalase; Gpx, Glutathione peroxidase; MDA, Malondialdehyde; OS, oxidative stress; ROS, reactive oxygen species.

The way diet, nutrients, and/or nutritional deficiencies can cause schizophrenia or exacerbate its symptoms is still a subject of research. However, various hypotheses have been proposed. These consist of factors such as inadequate diet, harmful eating habits, and/or nutritional deficiencies associated with the occurrence of hyperhomocysteinemia, oxidative-antioxidative imbalance, impaired immune system, and variations in levels of pro-inflammatory markers. The neuroprogressive hypothesis has highlighted the importance of oxidative stress and inflammation in schizophrenia, suggesting that these changes may represent possible mechanisms in the progression of the disease (3). To better understand these complex relationships, Figure 6 below illustrates how nutritional deficiencies can influence the levels of ROS and exacerbate schizophrenia symptoms. The effects of nutritional deficiencies on oxidative stress are highlighted, emphasizing the possible mechanisms by which these deficiencies contribute to disease progression. Research has demonstrated that the disruption of the oxidative stress system can cause obesity by altering diet and promoting fat accumulation in the body (177).

Pro-inflammatory cytokines, microglial cells, astrocytes, and immune cells such as monocytes, macrophages, and T or B lymphocytes are involved in controlling inflammation in the central nervous system. A balanced inflammatory process is crucial for tissue health, but excessive inflammatory reactions can cause additional cellular damage. Uncontrolled inflammation can arise from infectious agents (such as bacteria or viruses), a reaction to neuronal injury from trauma, genetic defects, or environmental toxins (178). In the central nervous system, inflammation can have neuroprotective or neurotoxic effects depending on the interactions between genetic variations, environmental factors, and inflammatory responses. The latest study on inflammation and schizophrenia indicates that inflammation can increase the risk of schizophrenia and exacerbate its symptoms (59).

Inflammation is considered a risk factor for schizophrenia because it contributes to the pathophysiology and exacerbation of these conditions. Various factors, including diet, can control the level of inflammation (59). High-calorie foods, saturated fats, and simple carbohydrates can stimulate peripheral inflammatory markers, while foods rich in fiber and vegetables reduce inflammation (15). Research has examined the role of polyunsaturated fats in psychosis. Two polyunsaturated fats are highlighted in the literature: essential fatty acids omega-3 and omega-6. Omega-6 fatty acids are pro-inflammatory, while omega-3 fatty acids are anti-inflammatory (179).

Accumulated information from previous research shows that inflammation has been identified as a factor in the development of schizophrenia (180). In addition to the two important players in neuroinflammation, pro-inflammatory cytokines and microglial activation, a diet that causes inflammation exacerbates neuroinflammation by promoting the pathophysiological changes of schizophrenia and the severity of symptoms. Considering the impact that dietary regimes have on inflammatory markers, we emphasize the importance of nutrients such as omega-3 polyunsaturated fatty acids, vitamin D, and vitamin B in the treatment of schizophrenia symptoms (59).

A gut-brain connection should be examined regarding the link between dietary inflammation and schizophrenia. Changes in the composition of the microbiota and microbial metabolites influence gut health and immune system reactions. The gut imbalance has been noted in patients with schizophrenia, and the increased presence of certain types of bacteria, *Succinivibrio* and *Corynebacterium*, has been significantly correlated with the severity of schizophrenia symptoms (181). Furthermore, recent studies have reported that patients with schizophrenia exhibit increased intestinal permeability, often referred to as “leaky gut” (182). If this permeability persists over time, it can compromise the blood–brain barrier, allowing harmful substances to enter the brain and potentially damage neurons. This disruption may exacerbate symptoms and accelerate the onset of schizophrenia (183). Moreover, patients with schizophrenia exhibit significant imbalances in gut flora compared to healthy individuals. Research indicates that specific bacterial populations are altered in these patients, which may contribute to their psychiatric symptoms (184, 185). For instance, disturbances in the gut microbiome can affect neurotransmitter metabolism, including glutamate and GABA, which are critical for maintaining mental health (186). Additionally, animal studies have shown that manipulation of gut flora through fecal transplants can lead to schizophrenia-like behavioral changes, further supporting the connection between gut health and mental health outcomes (182, 184). These findings underscore the importance of considering gastrointestinal health in understanding the pathophysiology of schizophrenia. Gut imbalance can influence susceptibility to infections and inflammation, leading to the acceleration of schizophrenia onset and symptom intensification. It is believed that dietary substances considered in schizophrenia exert their effects at least partially through the gut-brain axis (59).

The advice to consume anti-inflammatory nutrients or foods as part of a balanced diet has long-term benefits in reducing the side effects of medications. As a result, substances with anti-inflammatory properties are beneficial in reducing the severity of disease symptoms and decreasing risk factors that may trigger schizophrenia (59). Vegetables, especially those untouched by chemicals or technological processes, contain high levels of antioxidant compounds that can counteract the harmful effects of redox processes in our bodies (187).

## 6 Limitations

The field of nutritional psychiatry is a new one, and there is consensus about the specific impact of diet and nutritional deficiencies on schizophrenia, suggesting that further studies are needed. Also, most of the studies included in this review are cross-sectional and do not follow participants long-term, which limits understanding of the cumulative effects of nutrition on mental health. Moreover, exposure to stress, lifestyle, stress, and other factors can influence both diet and the risk of schizophrenia. The methods of assessing diet and nutritional status vary from one study to another, this may introduce the risk of errors and may affect the accuracy of the results.

## 7 Conclusion

Schizophrenia is influenced by both genetic and environmental factors, including diet. Nutritional deficiencies, such as lack of omega-3 fatty acids, vitamin D, and B vitamins, during the prenatal and postnatal periods, can negatively impact neurodevelopment and increase the risk of schizophrenia. Schizophrenia patients show antioxidant enzyme imbalances and reduced levels of antioxidants, leading to increased markers of oxidative stress. Additionally, cytokine-mediated inflammation, microglial activation, and gut dysbiosis are associated with schizophrenia onset and symptom severity. Currently, there is no universally accepted diet for the treatment of schizophrenia. However, different diets and nutritional methods, such as the Mediterranean diet, the ketogenic diet, the gluten-free diet, and the DASH diet, are being researched and applied to alleviate symptoms and improve overall health. A healthy diet rich in anti-inflammatory and antioxidant nutrients can help manage schizophrenia by reducing oxidative stress, preventing complications, and improving quality of life. Given the essential role of omega-3 fatty acids in brain development and function, their deficiency has been linked to an increased risk of schizophrenia. Similarly, vitamin D deficiency is prevalent among individuals with schizophrenia and correlates with symptom severity; thus, maintaining adequate levels of vitamin D is crucial for brain development and function. B vitamins, especially folic acid, vitamin B6, and vitamin B12, play an essential role in cellular metabolism and neurotransmission. Deficiencies of these vitamins are also associated with a heightened risk of developing schizophrenia.

As our understanding of the relationship between diet, nutrition, and schizophrenia evolves, tailored dietary interventions are likely to become an increasingly important component of comprehensive treatment plans for individuals with schizophrenia. Future research should focus on identifying specific dietary patterns and nutrient combinations that effectively reduce symptoms and improve outcomes for people with schizophrenia. Additionally, clinical trials evaluating the combined effects of multiple nutrients, such as omega-3-fatty acids, vitamin D, and B vitamins, should be prioritized to establish evidence-based dietary recommendations.

In summary, integrating nutritional strategies into the management of schizophrenia holds promise for enhancing patient care. Continued exploration of the links between food, nutrition, gut microbiome profiles, and mental health will be essential for developing effective interventions aimed at improving the lives of those affected by schizophrenia.

## Data Availability

The original contributions presented in the study are included in the article/supplementary material, further inquiries can be directed to the corresponding author/s.

## References

[1] Jacka FN Mykletun A Berk M. Moving towards a population health approach to the primary prevention of common mental disorders. BMC Med. (2012):10. doi: 10.1186/1741-7015-10-149 23186355 PMC3534562

[2] Penninx BWJH Lange SMM. Metabolic syndrome in psychiatric patients: overview, mechanisms, and implications. Dialogues Clin Neurosci. (2018) 20:63–73. doi: 10.31887/DCNS.2018.20.1/BPENNINX, PMID: 29946213 PMC6016046

[3] Onaolapo OJ Onaolapo AY. Nutrition, nutritional deficiencies, and schizophrenia: an association worthy of constant reassessment. World J Clin Cases. (2021) 9:8295–311. doi: 10.12998/wjcc.v9.i28.8295, PMID: 34754840 PMC8554424

[4] Morera-Fumero AL Abreu-Gonzalez P. Role of melatonin in schizophrenia. Int J Mol Sci. (2013) 14:9037–50. doi: 10.3390/ijms14059037, PMID: 23698762 PMC3676771

[5] Tang PY Tee SF Su KP. Editorial: the link between nutrition and schizophrenia. Front Psych. (2022) 13:120. doi: 10.3389/fpsyt.2022.1074120, PMID: 36479557 PMC9720389

[6] El Kirat H Khattabi A Khalis M Belrhiti Z. Effects of physical activity and nutrient supplementation on symptoms and well-being of schizophrenia patients: an umbrella review. Schizophr Res. (2023) 262:112–20. doi: 10.1016/j.schres.2023.10.021, PMID: 37948884

[7] Velligan DI Rao S. The epidemiology and global burden of schizophrenia. J Clin Psychiatry. (2023) 84:5. doi: 10.4088/JCP.MS21078COM5 36652681

[8] Charlson FJ Ferrari AJ Santomauro DF Diminic S Stockings E Scott JG . Global epidemiology and burden of schizophrenia: findings from the global burden of disease study 2016. Schizophr Bull. (2018) 44:1195–203. doi: 10.1093/schbul/sby058, PMID: 29762765 PMC6192504

[9] Li X Wei N Song J Liu J Yuan J Song R . The global burden of schizophrenia and the impact of urbanization during 1990–2019: an analysis of the global burden of disease study 2019. Environ Res. (2023):232. doi: 10.1016/j.envres.2023.116305 37268204

[10] Solmi M Seitidis G Mavridis D Correll CU Dragioti E Guimond S . Incidence, prevalence, and global burden of schizophrenia - data, with critical appraisal, from the global burden of disease (GBD) 2019. Mol Psychiatry. (2023) 28:5319–27. doi: 10.1038/s41380-023-02138-4, PMID: 37500825

[11] Jauhar S Laws K Fusar-Poli P McKenna P. Relapse prevention in schizophrenia. The Lancet Psychiatry. (2022) 9:e13. doi: 10.1016/S2215-0366(21)00501-0 35305747

[12] Suvisaari J Keinänen J Eskelinen S Mantere O. Diabetes and Schizophrenia. Curr Diab Rep. (2016) 16. doi: 10.1016/S2215-0366(21)00501-0 26803652

[13] Patel KR Cherian J Gohil K Atkinson D. Schizophrenia: Overview and treatment options. P and T. (2014) 39:638–45. PMID: 25210417 PMC4159061

[14] Stępnicki P Kondej M Kaczor AA. Current concepts and treatments of schizophrenia. Molecules. (2018) 23:2087. doi: 10.3390/molecules23082087 30127324 PMC6222385

[15] Firth J Veronese N Cotter J Shivappa N Hebert JR Ee C . What is the role of dietary inflammation in severe mental illness? A review of observational and experimental findings. Front Psych. (2019) 10:350. doi: 10.3389/fpsyt.2019.00350, PMID: 31156486 PMC6529779

[16] Sarris J. Nutritional psychiatry: from concept to the clinic. Drugs. (2019) 79:929–34. doi: 10.1007/s40265-019-01134-9, PMID: 31114975

[17] Owen L Corfe B. The role of diet and nutrition on mental health and wellbeing. Proc Nutr Soc. (2017) 76:425–6. doi: 10.1017/S0029665117001057 28707609

[18] Zhang R Zhang B Shen C Sahakian BJ Li Z Zhang W . Associations of dietary patterns with brain health from behavioral, neuroimaging, biochemical and genetic analyses. Nat Ment Health. (2024) 2:535–52. doi: 10.1038/s44220-024-00226-0

[19] Gresham E Bisquera A Byles JE Hure AJ. Effects of dietary interventions on pregnancy outcomes: a systematic review and meta-analysis. Matern Child Nutr. (2016) 12:5–23. doi: 10.1111/mcn.12142, PMID: 25048387 PMC6860081

[20] Zhu Y Hedderson MM Sridhar S Xu F Feng J Ferrara A. Poor diet quality in pregnancy is associated with increased risk of excess fetal growth: a prospective multi-racial/ethnic cohort study. Int J Epidemiol. (2019) 48:423–32. doi: 10.1093/ije/dyy285, PMID: 30590563 PMC6469312

[21] Fitzgerald E Hor K Drake AJ. Maternal influences on fetal brain development: the role of nutrition, infection and stress, and the potential for intergenerational consequences. Early Hum Dev. (2020) 150:105190. doi: 10.1016/j.earlhumdev.2020.105190, PMID: 32948364 PMC7481314

[22] Kirkbride JB Susser E Kundakovic M Kresovich JK Davey Smith G Relton CL. Prenatal nutrition, epigenetics and schizophrenia risk: can we test causal effects? Epigenomics. (2012) 4:303–15. doi: 10.2217/epi.12.20, PMID: 22690666 PMC3970193

[23] Rudzki L Stone TW Maes M Misiak B Samochowiec J Szulc A. Gut microbiota-derived vitamins – underrated powers of a multipotent ally in psychiatric health and disease. Prog Neuro-Psychopharmacol Biol Psychiatry. (2021) 107:110240. doi: 10.1016/j.pnpbp.2020.110240, PMID: 33428888

[24] Chaparro C Damasio A. The feeling of what happens body and emotion in the making of consciousness. New York: Harcourt Brace and Company (1999). 386 p.

[25] Peet M. Diet, diabetes and schizophrenia: review and hypothesis. Br J Psychiatry. (2004) 184:s102–5. doi: 10.1192/bjp.184.47.s102, PMID: 15056602

[26] Elman I Borsook D Lukas SE. Food intake and reward mechanisms in patients with schizophrenia: implications for metabolic disturbances and treatment with second-generation antipsychotic agents. Neuropsychopharmacology. (2006) 31:2091–120. doi: 10.1038/sj.npp.1301051, PMID: 16541087

[27] Schwarzenberg SJ Georgieff MK Daniels S Corkins M Golden NH Kim JH . Advocacy for improving nutrition in the first 1000 days to support childhood development and adult health. Pediatrics. (2018) 141:3716. doi: 10.1542/peds.2017-3716, PMID: 29358479

[28] Enderami A Zarghami M Darvishi-Khezri H. The effects and potential mechanisms of folic acid on cognitive function: a comprehensive review. Neurol Sci. (2018) 39:1667–75. doi: 10.1007/s10072-018-3473-4 29936555

[29] Maekawa M Watanabe A Iwayama Y Kimura T Hamazaki K Balan S . Polyunsaturated fatty acid deficiency during neurodevelopment in mice models the prodromal state of schizophrenia through epigenetic changes in nuclear receptor genes. Transl Psychiatry. (2017) 7:e1229. doi: 10.1038/tp.2017.182, PMID: 28872641 PMC5639238

[30] Brown AS Derkits EJ. Prenatal infection and schizophrenia: a review of epidemiologic and translational studies. Am J Psychiatry. (2010) 167:261–80. doi: 10.1176/appi.ajp.2009.09030361 20123911 PMC3652286

[31] Kwon HK Choi GB Huh JR. Maternal inflammation and its ramifications on fetal neurodevelopment. Trends Immunol. (2022) 43:230–44. doi: 10.1016/j.it.2022.01.007 35131181 PMC9005201

[32] Cryan JF O’Riordan KJ Sandhu K Peterson V Dinan TG. The gut microbiome in neurological disorders. Lancet Neurol. Lancet Neurol. (2020) 19:179–94. doi: 10.1016/S1474-4422(19)30356-4 31753762

[33] Amir M Brown JA Rager SL Sanidad KZ Ananthanarayanan A Zeng MY. Maternal microbiome and infections in pregnancy. Microorganisms. (2020) 8:1996. doi: 10.3390/microorganisms8121996 33333813 PMC7765218

[34] Shen W Qiu W Liu Y Liao W Ma Y He Y . Postnatal age is strongly correlated with the early development of the gut microbiome in preterm infants. Transl Pediatr. (2021) 10:2313–24. doi: 10.21037/tp-21-367, PMID: 34733672 PMC8506066

[35] Stiemsma LT Michels KB. The role of the microbiome in the developmental origins of health and disease. Pediatrics. (2018) 141:2437. doi: 10.1542/peds.2017-2437, PMID: 29519955 PMC5869344

[36] Yao Y Cai X Ye Y Wang F Chen F Zheng C. The role of microbiota in infant health: from early life to adulthood. Front Immunol. (2021) 12:472. doi: 10.3389/fimmu.2021.708472 PMC8529064 34691021

[37] Marshall NE Abrams B Barbour LA Catalano P Christian P Friedman JE . The importance of nutrition in pregnancy and lactation: lifelong consequences. Am J Obstet Gynecol. (2022) 226:607–32. doi: 10.1016/j.ajog.2021.12.035 34968458 PMC9182711

[38] Catassi G Aloi M Giorgio V Gasbarrini A Cammarota G Ianiro G. The role of diet and nutritional interventions for the infant gut microbiome. Nutrients. (2024) 16:400. doi: 10.3390/nu16030400 38337684 PMC10857663

[39] Maher SE O’Brien EC Moore RL Byrne DF Geraghty AA Saldova R . The association between the maternal diet and the maternal and infant gut microbiome: a systematic review. Br J Nutr. (2023) 129:1491–9. doi: 10.1017/S0007114520000847, PMID: 32129734

[40] Abdul QA Yu BP Chung HY Jung HA Choi JS. Epigenetic modifications of gene expression by lifestyle and environment. Arch Pharm Res. (2017) 40:1219–37. doi: 10.1007/s12272-017-0973-3 29043603

[41] Mierziak J Kostyn K Boba A Czemplik M Kulma A Wojtasik W. Influence of the bioactive diet components on the gene expression regulation. Nutrients. (2021) 13:3673. doi: 10.3390/nu13113673 34835928 PMC8619229

[42] McGrath J Brown A St Clair D. Prevention and schizophrenia - the role of dietary factors. Schizophr Bull. (2011) 37:272–83. doi: 10.1093/schbul/sbq121, PMID: 20974747 PMC3044637

[43] Gasmi A Nasreen A Menzel A Gasmi Benahmed A Pivina L Noor S . Neurotransmitters regulation and food intake: the role of dietary sources in neurotransmission. Molecules. (2023) 28:210. doi: 10.3390/molecules28010210 PMC9822089 36615404

[44] Lakhan SE Vieira KF. Nutritional therapies for mental disorders. Nutr J. (2008) 7:2. doi: 10.1186/1475-2891-7-2 18208598 PMC2248201

[45] Davis J Eyre H Jacka FN Dodd S Dean O McEwen S . A review of vulnerability and risks for schizophrenia: beyond the two hit hypothesis. Neurosci Biobehav Rev. (2016) 65:185–94. doi: 10.1016/j.neubiorev.2016.03.017, PMID: 27073049 PMC4876729

[46] Cernigliaro F Santangelo A Nardello R Lo Cascio S D’Agostino S Correnti E . Prenatal nutritional factors and neurodevelopmental disorders: a narrative review. Life. (2024) 14:1084. doi: 10.3390/LIFE14091084, PMID: 39337868 PMC11433086

[47] Sullivan EL Nousen EK Chamlou KA. Maternal high fat diet consumption during the perinatal period programs offspring behavior. Physiol Behav. (2014) 123:236–42. doi: 10.1016/j.physbeh.2012.07.014 23085399 PMC3594403

[48] Ortiz-Valladares M Gonzalez-Perez O Pedraza-Medina R. Bridging the gap: prenatal nutrition, myelination, and schizophrenia etiopathogenesis. Neuroscience. (2024) 558:58–69. doi: 10.1016/J.NEUROSCIENCE.2024.08.019 39159841

[49] Georgieff MK Ramel SE Cusick SE. Nutritional influences on brain development. Acta Paediatr. (2018) 107:1310–21. doi: 10.1111/apa.14287 29468731 PMC6045434

[50] Jenkins TA. Perinatal complications and schizophrenia: involvement of the immune system. Front Neurosci. (2013) 7:110. doi: 10.3389/fnins.2013.00110 23805069 PMC3691516

[51] Saidi L Hammou H Sicard F Landrier JF Mounien L. Maternal vitamin D deficiency and brain functions: A never-ending story. Food Funct. (2023) 14:6290–301. doi: 10.1039/d3fo00166k 37350315

[52] Crews M Lally J Gardner-Sood P Howes O Bonaccorso S Smith S . Vitamin D deficiency in first episode psychosis: a case-control study. Schizophr Res. (2013) 150:533–7. doi: 10.1016/j.schres.2013.08.036, PMID: 24060571

[53] Wrzosek M Lukaszkiewicz J Wrzosek M Jakubczyk A Matsumoto H Piatkiewicz P . Vitamin D and the central nervous system. Pharmacol Rep. (2013) 65:271–8. doi: 10.1016/s1734-1140(13)71003-x 23744412

[54] Eyles DW. Vitamin D: Brain and behavior. JBMR Plus. (2021) 5:e10419. doi: 10.1002/jbm4.10419 33553986 PMC7839822

[55] Neriman A Hakan Y Ozge U. The psychotropic effect of vitamin D supplementation on schizophrenia symptoms. BMC Psychiatry. (2021) 21:3308. doi: 10.1186/s12888-021-03308-w PMC8204117 34130647

[56] Yüksel RN Altunsoy N Tikir B Cingi Külük M Unal K Goka S . Correlation between total vitamin D levels and psychotic psychopathology in patients with schizophrenia: therapeutic implications for add-on vitamin D augmentation. Ther Adv Psychopharmacol. (2014) 4:268–75. doi: 10.1177/2045125314553612, PMID: 25489478 PMC4257987

[57] Lally J Gaughran F. Vitamin D in schizophrenia and depression: a clinical review. BJPsych Adv. (2019) 25:240–8. doi: 10.1192/bja.2019.10

[58] Schoenrock SA Tarantino LM. Developmental vitamin D deficiency and schizophrenia: The role of animal models. Genes Brain Behav. (2016) 15:45–61. doi: 10.1111/gbb.12271 26560996 PMC4768902

[59] Cha HY Yang SJ. Anti-inflammatory diets and schizophrenia. Nutr Res. (2020) 9:241–57. doi: 10.7762/cnr.2020.9.4.241, PMID: 33204665 PMC7644368

[60] Calvo MS Whiting SJ Barton CN. Vitamin D intake: a global perspective of current status. J Nutr. (2005) 135:310–6. doi: 10.1093/jn/135.2.310 15671233

[61] Wassif GA Alrehely MS Alharbi DM Aljohani AA. The impact of vitamin D on neuropsychiatric disorders. Cureus. (2023) 15:e47716. doi: 10.7759/cureus.47716, PMID: 38022259 PMC10676226

[62] Hewison M Bouillon R Giovannucci E Goltzman D Meyer M Welsh JE. Feldman and Pike’s vitamin D: volume two: disease and therapeutics. Amsterdam, Netherlands: Elsevier (2023).

[63] Cui X McGrath JJ Burne THJ Eyles DW. Vitamin D and schizophrenia: 20 years on. Mol Psychiatry. (2021) 26:2708–20. doi: 10.1038/s41380-021-01025-0 33500553 PMC8505257

[64] van der Leeuw C de Witte LD Stellinga A van der Ley C Bruggeman R Kahn RS . Vitamin D concentration and psychotic disorder: associations with disease status, clinical variables and urbanicity. Psychol Med. (2020) 50:1680–6. doi: 10.1017/S0033291719001739, PMID: 31327333

[65] Fryar-Williams S Strobel J Clements P. Molecular mechanisms provide a landscape for biomarker selection for schizophrenia and schizoaffective psychosis. Int J Mol Sci. (2023) 24:296. doi: 10.3390/ijms242015296, PMID: 37894974 PMC10607016

[66] García-Miss M d R Pérez-Mutul J López-Canul B Solís-Rodríguez F Puga-Machado L Oxté-Cabrera A . Folate, homocysteine, interleukin-6, and tumor necrosis factor alfa levels, but not the methylenetetrahydrofolate reductase C677T polymorphism, are risk factors for schizophrenia. J Psychiatr Res. (2010) 44:441–6. doi: 10.1016/j.jpsychires.2009.10.011 19939410

[67] Bouaziz N Ayedi I Sidhom O Kallel A Rafrafi R Jomaa R . Plasma homocysteine in schizophrenia: determinants and clinical correlations in Tunisian patients free from antipsychotics. Psychiatry Res. (2010) 179:24–9. doi: 10.1016/j.psychres.2010.04.008, PMID: 20471108

[68] Cao B Sun XY Zhang CB Yan JJ Zhao QQ Yang SY . Association between B vitamins and schizophrenia: a population-based case-control study. Psychiatry Res. (2018) 259:501–5. doi: 10.1016/j.psychres.2017.11.006, PMID: 29154172

[69] Susser E St Clair D. Prenatal famine and adult mental illness: interpreting concordant and discordant results from the Dutch and Chinese famines. Soc Sci Med. (2013) 97:325–30. doi: 10.1016/j.socscimed.2013.02.049, PMID: 23557675

[70] Jose R Venketeswaramurthy N Sambath KR. A critical review on hypothesis, pathophysiology of schizophrenia, and role of vitamins in its management. Asian J Pharm Clin Res. (2018) 11:25. doi: 10.22159/ajpcr.2018.v11i8.23259

[71] Eyles D Burne T Mcgrath J. Vitamin D in fetal brain development. Semin Cell Dev Biol. (2011) 22:629–36. doi: 10.1016/j.semcdb.2011.05.004 21664981

[72] Brown AS Susser ES. Prenatal nutritional deficiency and risk of adult schizophrenia. Schizophr Bull. (2008) 34:1054–63. doi: 10.1093/schbul/sbn096, PMID: 18682377 PMC2632499

[73] Hoffer LJ. Vitamin therapy in schizophrenia. Isr J Psychiatry Relat Sci. (2008) 45:3–10. PMID: 18587164

[74] Freedman R Hunter SK Law AJ Clark AM Roberts A Hoffman MC. Choline, folic acid, vitamin D, and fetal brain development in the psychosis spectrum. Schizophr Res. (2022) 247:16–25. doi: 10.1016/j.schres.2021.03.008, PMID: 33838984 PMC8494861

[75] Insel BJ Schaefer CA McKeague IW Susser ES Brown AS. Maternal iron deficiency and the risk of schizophrenia in offspring. Arch Gen Psychiatry. (2008) 65:1136–44. doi: 10.1001/archpsyc.65.10.1136, PMID: 18838630 PMC3656467

[76] Sørensen HJ Nielsen PR Pedersen CB Mortensen PB. Association between prepartum maternal iron deficiency and offspring risk of schizophrenia: population-based cohort study with linkage of danish national registers. Schizophr Bull. (2011) 37:167. doi: 10.1093/schbul/sbp167 PMC3160221 20093425

[77] Xu J He G Zhu J Zhou X Clair DS Wang T . Prenatal nutritional deficiency reprogrammed postnatal gene expression in mammal brains: implications for schizophrenia. Int J Neuropsychopharmacol. (2015) 18:1–9. doi: 10.1093/IJNP/PYU054 PMC4360220 25522397

[78] Labrie V Lipina T Roder JC. Mice with reduced NMDA receptor glycine affinity model some of the negative and cognitive symptoms of schizophrenia. Psychopharmacology. (2008) 200:217–30. doi: 10.1007/s00213-008-1196-6, PMID: 18597079

[79] Zajkowska I Niczyporuk P Urbaniak A Tomaszek N Modzelewski S Waszkiewicz N. Investigating the impacts of diet, supplementation, microbiota, gut–brain Axis on schizophrenia: a narrative review. Nutrients. (2024) 16:2228. doi: 10.3390/NU16142228 39064675 PMC11279812

[80] Susser E Neugebauer R Hoek HW Brown AS Lin S Labovitz D . Schizophrenia after prenatal famine further evidence. Arch Gen Psychiatry. (1996) 53:25–31. doi: 10.1001/archpsyc.1996.01830010027005, PMID: Retraction in: Arch Gen Psychiatry. 1997 Jun;54(6):577-8. doi: 10.1001/archpsyc.1997.01830180095015 8540774

[81] Roffma RJL Lamberti S Achtyes E Macklin EA Galendez G Raeke L . A multi-center investigation of folate plus B12 supplementation in schizophrenia. Neuropsychopharmacology. (2011) 36:481–9. doi: 10.1001/jamapsychiatry.2013.900 PMC4394629 23467813

[82] Lewis CJ Crane NT Wilson DB Yetley EA. Estimated folate intakes: data updated to reflect food fortification, increased bioavailability, and dietary supplement use. Am J Clin Nutr. (1999) 70:198–207. doi: 10.1093/ajcn.70.2.198, PMID: 10426695

[83] Kennedy DO. B vitamins and the brain: mechanisms, dose and efficacy—a review. Nutrients. (2016) 8:68. doi: 10.3390/nu8020068 26828517 PMC4772032

[84] Bourre J-M. Acides gras ω-3 et troubles psychiatriques. Sciences. (2005) 21:216–21. doi: 10.1051/medsci/2005212216, PMID: 15691497

[85] Häfner H Nowotny B. Epidemiology of early-onset schizophrenia. Eur Arch Psychiatry Clin Neurosci. (1995) 245:80–92. doi: 10.1007/BF02190734 7654792

[86] Hsu MC Huang YS Ouyang WC. Beneficial effects of omega-3 fatty acid supplementation in schizophrenia: possible mechanisms. Lipids Health Dis. (2020) 19:159. doi: 10.1186/s12944-020-01337-0, PMID: 32620164 PMC7333328

[87] Van Erp TGM Hibar DP Rasmussen JM Glahn DC Pearlson GD Andreassen OA . Subcortical brain volume abnormalities in 2028 individuals with schizophrenia and 2540 healthy controls via the ENIGMA consortium. Mol Psychiatry. (2016) 21:547–53. doi: 10.1038/mp.2015.63, PMID: 26033243 PMC4668237

[88] Bruce KD Zsombok A Eckel RH. Lipid processing in the brain: a key regulator of systemic metabolism. Front Endocrinol. (2017) 8:60. doi: 10.3389/fendo.2017.00060, PMID: 28421037 PMC5378716

[89] Mocking RJT Assies J Ruhé HG Schene AH. Focus on fatty acids in the neurometabolic pathophysiology of psychiatric disorders. J Inherit Metab Dis. (2018) 41:597–611. doi: 10.1007/s10545-018-0158-3, PMID: 29524021

[90] Hedelin M Löf M Olsson M Lewander T Nilsson B Hultman CM . Dietary intake of fish, omega-3, omega-6 polyunsaturated fatty acids and vitamin D and the prevalence of psychotic-like symptoms in a cohort of 33 000 women from the general population. BMC Psychiatry. (2010) 10:10. doi: 10.1186/1471-244X-10-38, PMID: 20504323 PMC2889879

[91] Lange KW. Omega-3 fatty acids and mental health. Glob Health J. (2020) 4:18–30. doi: 10.1016/j.glohj.2020.01.004

[92] Amminger GP Schäfer MR Schlögelhofer M Klier CM McGorry PD. Longer-term outcome in the prevention of psychotic disorders by the Vienna omega-3 study. Nat Commun. (2015) 6:7934. doi: 10.1038/ncomms8934, PMID: 26263244 PMC4918317

[93] Bozzatello P De Rosa ML Rocca P Bellino S. Effects of omega 3 fatty acids on main dimensions of psychopathology. Int J Mol Sci. (2020) 21:42. doi: 10.3390/ijms21176042, PMID: 32839416 PMC7504659

[94] Bourre JM Faivre A Dumont O Nouvelot A Loudes C Puymirat J . Effect of polyunsaturated fatty acids on fetal mouse brain cells in culture in a chemically defined medium. J Neurochem. (1983) 41:1234–42. doi: 10.1111/j.1471-4159.1983.tb00817.x, PMID: 6619863

[95] Bourre JM Pascal G Durand G Masson M Dumont O Piciotti M. Alterations in the fatty acid composition of rat brain cells (neurons, astrocytes, and oligodendrocytes) and of subcellular fractions (myelin and Synaptosomes) induced by a diet devoid of n-3 fatty acids. J Neurochem. (1984) 43:342–8. doi: 10.1111/j.1471-4159.1984.tb00906.x, PMID: 6736955

[96] Blasbalg TL Hibbeln JR Ramsden CE Majchrzak SF Rawlings RR. Changes in consumption of omega-3 and omega-6 fatty acids in the United States during the 20th century. Am J Clin Nutr. (2011) 93:950–62. doi: 10.3945/ajcn.110.006643, PMID: 21367944 PMC3076650

[97] Caffrey A McNulty H Irwin RE Walsh CP Pentieva K. *Maternal folate nutrition and offspring health: Evidence and current controversies*. In: Proceedings of the Nutrition Society. (2019). 10.1017/S0029665118002689 30585558

[98] Cusick SE Georgieff MK. The role of nutrition in brain development: the Golden opportunity of the “first 1000 days.”. J Pediatr. (2016) 175:16–21. doi: 10.1016/j.jpeds.2016.05.013, PMID: 27266965 PMC4981537

[99] Fleishhacker WW Cetkovich-Bakmas M de Hert M Hennekens CH Lambert M Leucht S . Comorbid somatic illnesses in patients with severe mental disorders. Dis Nerv Syst. (2008) 69:514–9. doi: 10.4088/JCP.v69n0401 18370570

[100] Maayan L Correll CU. Management of antipsychotic-related weight gain. Expert Rev Neurother. (2010) 10:1175–200. doi: 10.1586/ern.10.85, PMID: 20586697 PMC3501406

[101] Dayabandara M Hanwella R Ratnatunga S Seneviratne S Suraweera C de Silva VA. Antipsychotic-associated weight gain: management strategies and impact on treatment adherence. Neuropsychiatr Dis Treat. (2017) 13:2231–41. doi: 10.2147/NDT.S113099, PMID: 28883731 PMC5574691

[102] De Hert M Detraux J Van Winkel R Yu W Correll CU. Metabolic and cardiovascular adverse effects associated with antipsychotic drugs. Nat Rev Endocrinol. (2012) 8:114–26. doi: 10.1038/nrendo.2011.156 22009159

[103] Holt RIG. The Management of Obesity in people with severe mental illness: an unresolved conundrum. Psychother Psychosom. (2019) 88:327–32. doi: 10.1159/000503835, PMID: 31587002

[104] Misiak B Piotrowski P Beszłej JA Kalinowska S Chęć M Samochowiec J. Metabolic dysregulation and psychosocial stress in patients with schizophrenia spectrum disorders: a case-control study. J Clin Med. (2020) 9:822. doi: 10.3390/jcm9123822, PMID: 33255883 PMC7760156

[105] Harris LW Guest PC Wayland MT Umrania Y Krishnamurthy D Rahmoune H . Schizophrenia: metabolic aspects of aetiology, diagnosis and future treatment strategies. Psychoneuroendocrinology. (2013) 38:752–66. doi: 10.1016/j.psyneuen.2012.09.009, PMID: 23084727

[106] Lee J Xue X Au E McIntyre WB Asgariroozbehani R Panganiban K . Glucose dysregulation in antipsychotic-naive first-episode psychosis: in silico exploration of gene expression signatures. Transl Psychiatry. (2024) 14:716. doi: 10.1038/s41398-023-02716-8 PMC10781725 38199991

[107] Pillinger T McCutcheon RA Vano L Mizuno Y Arumuham A Hindley G . Comparative effects of 18 antipsychotics on metabolic function in patients with schizophrenia, predictors of metabolic dysregulation, and association with psychopathology: a systematic review and network meta-analysis. Lancet Psychiatry. (2020) 7:64–77. doi: 10.1016/S2215-0366(19)30416-X, PMID: 31860457 PMC7029416

[108] Papanastasiou E. The prevalence and mechanisms of metabolic syndrome in schizophrenia: a review. Ther Adv Psychopharmacol. (2013) 3, 3:33–51. doi: 10.1177/2045125312464385 23983991 PMC3736963

[109] Misiak B Łaczmański Ł Słoka NK Szmida E Piotrowski P Loska O . Metabolic dysregulation in first-episode schizophrenia patients with respect to genetic variation in one-carbon metabolism. Psychiatry Res. (2016):238. doi: 10.1016/j.psychres.2016.01.077 27086212

[110] Nicol GE Yingling MD Flavin KS Schweiger JA Patterson BW Schechtman KB . Metabolic effects of antipsychotics on adiposity and insulin sensitivity in youths: a randomized clinical trial. JAMA Psychiatry Am Med Association. (2018) 75:788–96. doi: 10.1001/JAMAPSYCHIATRY.2018.1088 PMC6143095 29898210

[111] Teff KL Rickels MR Grudziak J Fuller C Nguyen HL Rickels K. Antipsychotic-induced insulin resistance and postprandial hormonal dysregulation independent of weight gain or psychiatric disease. Diabetes. (2013) 62:3232–40. doi: 10.2337/DB13-0430/-/DC1 23835329 PMC3749337

[112] Bernstein JG. Induction of obesity by psychotropic drugs. Ann N Y Acad Sci. (1987) 499:212. doi: 10.1111/j.1749-6632.1987.tb36212.x 2886102

[113] De Hert M Dekker JM Wood D Kahl KG Holt RIG Möller HJ. Cardiovascular disease and diabetes in people with severe mental illness position statement from the European psychiatric association (EPA), supported by the European Association for the Study of diabetes (EASD) and the European Society of Cardiology (ESC). Eur Psychiatry. (2009) 24:412–24. doi: 10.1016/j.eurpsy.2009.01.005 19682863

[114] Freudenthal R Moncrieff J. ‘A landmark in psychiatric progress’? The role of evidence in the rise and fall of insulin coma therapy. Hist Psychiatry. (2022) 33:65–78. doi: 10.1177/0957154X211062538, PMID: 34935541 PMC8886299

[115] Agarwal SM Caravaggio F Costa-Dookhan KA Castellani L Kowalchuk C Asgariroozbehani R . Brain insulin action in schizophrenia: something borrowed and something new. Neuropharmacology. (2020) 163:107633. doi: 10.1016/j.neuropharm.2019.05.010, PMID: 31077731

[116] de Bartolomeis A De Simone G De Prisco M Barone A Napoli R Beguinot F . Insulin effects on core neurotransmitter pathways involved in schizophrenia neurobiology: a meta-analysis of preclinical studies. Implications for the treatment. Mol Psychiatry. (2023) 28:2811–25. doi: 10.1038/s41380-023-02065-4 37085712 PMC10615753

[117] Naeem N Basit A Shiraz A Bin Zafar A Mustafa N Ali Siddique S . Insulin-associated weight gain in type 2 diabetes and its relation with caloric intake. Cureus. (2019) 11:e5275. doi: 10.7759/cureus.5275, PMID: 31576267 PMC6764643

[118] Ali SA Mathur N Malhotra AK Braga RJ. Electroconvulsive therapy and schizophrenia: a systematic review. Psychiatry. (2019) 5:75–83. doi: 10.1159/000497376, PMID: 31192220 PMC6528094

[119] Kantrowitz JT Correll CU Jain R Cutler AJ. New developments in the treatment of schizophrenia: an expert roundtable. Int J Neuropsychopharmacol. (2023) 26:322–30. doi: 10.1093/ijnp/pyad011, PMID: 36932673 PMC10229849

[120] Ban TA. Fifty years chlorpromazine: a historical perspective. Neuropsychiatr Dis Treat. (2007) 3:495–500. PMID: 19300578 PMC2655089

[121] Allison DB Mentore JL Heo M Chandler LP Cappelleri JC Infante MC . Antipsychotic-induced weight gain: a comprehensive research synthesis. Am J Psychiatry. (1999) 156:1686–96. doi: 10.1176/ajp.156.11.1686, PMID: 10553730

[122] Lu ML Wang TN Lin TY Shao WC Chang SH Chou JY . Differential effects of olanzapine and clozapine on plasma levels of adipocytokines and total ghrelin. Prog Neuro-Psychopharmacol Biol Psychiatry. (2015):58. doi: 10.1016/j.pnpbp.2014.12.001 25496829

[123] Ader M Kim SP Catalano KJ Ionut V Hucking K Richey JM . Metabolic dysregulation with atypical antipsychotics occurs in the absence of underlying disease: a placebo-controlled study of olanzapine and risperidone in dogs. Diabetes. (2005) 54:862–71. doi: 10.2337/diabetes.54.3.862, PMID: 15734866

[124] Nasrallah HA Newcomer JW. Atypical antipsychotics and metabolic dysregulation. J Clin Psychopharmacol. (2004) 24:S7–S14. doi: 10.1097/01.jcp.0000142282.62336.e9 15356415

[125] Silverstone T Smith G Goodall E. Prevalence of obesity in patients receiving depot antipsychotics. Br J Psychiatry. (1988) 153:214–7. doi: 10.1192/bjp.153.2.214, PMID: 2908236

[126] Kim M Yang SJ Kim HH Jo A Jhon M Lee JY . Effects of dietary habits on general and abdominal obesity in community-dwelling patients with schizophrenia. Clin Psychopharmacol Neurosci. (2023) 21:68–76. doi: 10.9758/cpn.2023.21.1.68, PMID: 36700313 PMC9889910

[127] Magalhães PVS Dean O Andreazza AC Berk M Kapczinski F. Antioxidant treatments for schizophrenia. Cochrane Database Syst Rev. (2016) 2016:CD008919. doi: 10.1002/14651858.CD008919.pub2, PMID: 26848926 PMC10337514

[128] Forman HJ Zhang H. Targeting oxidative stress in disease: promise and limitations of antioxidant therapy. Nat Rev Drug Discov. (2021) 20:689–709. doi: 10.1038/s41573-021-00233-1 34194012 PMC8243062

[129] Reddy R Reddy R. Antioxidant therapeutics for schizophrenia. Antioxid Redox Signal. (2011) 15:2047–55. doi: 10.1089/ars.2010.3571 20977337

[130] Hägg S Söderberg S Ahrén B Olsson T Mjörndal T. Leptin concentrations are increased in subjects treated with clozapine or conventional antipsychotics. Dis Nerv Syst. (2001) 62:843–8. doi: 10.4088/JCP.v62n1102 11775042

[131] Zabeau L Lavens D Peelman F Eyckerman S Vandekerckhove J Tavernier J. The ins and outs of leptin receptor activation. FEBS Lett. (2003) 546:45–50. doi: 10.1016/S0014-5793(03)00440-X, PMID: 12829235

[132] Dohan FC Grasberger JC Lowell FM Johnston HT Arbegast AW. Relapsed schizophrenics: more rapid improvement on a milk- and cereal-free diet. Br J Psychiatry. (1969) 115:595–6. doi: 10.1192/bjp.115.522.595, PMID: 5820122

[133] Tang M Zhao T Liu T Dang R Cai H Wang Y. Nutrition and schizophrenia: associations worthy of continued revaluation. Nutr Neurosci. Nutr Neurosci. (2024) 27:528–46. doi: 10.1080/1028415X.2023.2233176 37565574

[134] Krysta K Tredzbor B Martyniak E Piekarska-Bugiel K Koźmin-Burzyńska A Cieślik A . The correlation beteen lifestyle and risk of metabolic syndrome in schizophrenia. Eur Psychiatry. (2023) 66:S1066. doi: 10.1192/j.eurpsy.2023.2262

[135] Palmer CM Gilbert-Jaramillo J Westman EC. The ketogenic diet and remission of psychotic symptoms in schizophrenia: two case studies. Schizophr Res. (2019) 208:439–40. doi: 10.1016/j.schres.2019.03.019, PMID: 30962118

[136] Sethi S Wakeham D Ketter T Hooshmand F Bjornstad J Richards B . Ketogenic diet intervention on metabolic and psychiatric health in bipolar and schizophrenia: a pilot trial. Psychiatry res. Psychiatry Res. (2024) 335:115866. doi: 10.1016/J.PSYCHRES.2024.115866 38547601

[137] Kinsman SL Vining EPG Quaskey SA Mellits D Freeman JM. Efficacy of the ketogenic diet for intractable seizure disorders: review of 58 cases. Epilepsia. (1992) 33:1132–6. doi: 10.1111/j.1528-1157.1992.tb01770.x, PMID: 1464275

[138] Sarnyai Z Kraeuter AK Palmer CM. Ketogenic diet for schizophrenia: Clinical implication. Curr Opin Psychiatry. (2019) 32:394–401. doi: 10.1097/YCO.0000000000000535 31192814

[139] Kraeuter AK Loxton H Lima BC Rudd D Sarnyai Z. Ketogenic diet reverses behavioral abnormalities in an acute NMDA receptor hypofunction model of schizophrenia. Schizophr Res. (2015) 169:491–3. doi: 10.1016/j.schres.2015.10.041 26547882

[140] Włodarczyk A Wiglusz MS Cubała WJ. Ketogenic diet for schizophrenia: nutritional approach to antipsychotic treatment. Med Hypotheses. (2018):118. doi: 10.1016/j.mehy.2018.06.022 30037619

[141] Kraft BD Westman EC. Schizophrenia, gluten, and low-carbohydrate, ketogenic diets: a case report and review of the literature. Nutr Metab (Lond). (2009):6. doi: 10.1186/1743-7075-6-10 19245705 PMC2652467

[142] Kraeuter AK van den Buuse M Sarnyai Z. Ketogenic diet prevents impaired prepulse inhibition of startle in an acute NMDA receptor hypofunction model of schizophrenia. Schizophr Res. (2019):206. doi: 10.1016/j.schres.2018.11.011 30466960

[143] Kowalski K Bogudzińska B Stańczykiewicz B Piotrowski P Bielawski T Samochowiec J . The deficit schizophrenia subtype is associated with low adherence to the Mediterranean diet: findings from a case–control study. J Clin Med. (2022) 11:568. doi: 10.3390/jcm11030568 35160019 PMC8836983

[144] Madani S Ahmadi A Shoaei-Jouneghani F Moazen M Sasani N. The relationship between the Mediterranean diet and Axis I disorders: A systematic review of observational studies. Food Sci Nutr. (2022) 10:3241–58. doi: 10.1002/fsn3.2950 36249971 PMC9548357

[145] Tsigalou C Konstantinidis T Paraschaki A Stavropoulou E Voidarou C Bezirtzoglou E. Mediterranean diet as a tool to combat inflammation and chronic diseases. An overview. Biomedicines. (2020) 8:201. doi: 10.3390/biomedicines8070201 32650619 PMC7400632

[146] Esmaillzadeh A Zakizadeh E Faghihimani E Gohari M Jazayeri S. The effect of purslane seeds on glycemic status and lipid profiles of persons with type 2 diabetes: a randomized controlled cross-over clinical trial. J Res Med Sci. (2015) 20:47–53. PMID: 25767522 PMC4354065

[147] Trichopoulou A Costacou T Bamia C Trichopoulos D. Adherence to a Mediterranean diet and survival in a Greek population. N Engl J Med. (2003) 348:2599–608. doi: 10.1056/nejmoa025039 12826634

[148] Dobrosielski DA Papandreou C Patil SP Salas-Salvadó J. Diet and exercise in the management of obstructive sleep apnoea and cardiovascular disease risk. Eur Respir Rev. (2017) 26:160110. doi: 10.1183/16000617.0110-2016 28659501 PMC5559698

[149] Uddin MK Juraimi AS Hossain MS Nahar MAU Ali ME Rahman MM. Purslane weed (*Portulaca oleracea*): a prospective plant source of nutrition, omega-3 fatty acid, and antioxidant attributes. Sci World J. (2014) 2014:951019. doi: 10.1155/2014/951019 PMID: 24683365 PMC3934766

[150] Alam MA Juraimi AS Rafii MY Abdul Hamid A Aslani F Hasan MM . Evaluation of antioxidant compounds, antioxidant activities, and mineral composition of 13 collected purslane (*Portulaca oleracea* L.) accessions. Biomed Res Int. (2014) 2014:63. doi: 10.1155/2014/296063 PMC3918865 24579078

[151] Blumenthal JA Babyak MA Hinderliter A Watkins LL Craighead L Lin PH . Effects of the DASH diet alone and in combination with exercise and weight loss on blood pressure and cardiovascular biomarkers in men and women with high blood pressure: the ENCORE study. Arch Intern Med. (2010) 170:470. doi: 10.1001/archinternmed.2009.470 20101007 PMC3633078

[152] Larsson SC Wallin A Wolk A. Dietary approaches to stop hypertension diet and incidence of stroke. Stroke. (2016) 47:2675. doi: 10.1161/strokeaha.116.012675 26869384

[153] Miller ER Erlinger TP Young DR Jehn M Charleston J Rhodes D . Results of the diet, exercise, and weight loss intervention trial (DEW-IT). Hypertension. (2002) 40:2. doi: 10.1161/01.HYP.0000037217.96002.8E 12411452

[154] Sorić T Mavar M Rumbak I. The effects of the dietary approaches to stop hypertension (DASH) diet on metabolic syndrome in hospitalized schizophrenic patients: a randomized controlled trial. Nutrients. (2019) 11:950. doi: 10.3390/nu11122950 31817080 PMC6950694

[155] Firth J Stubbs B Sarris J Rosenbaum S Teasdale S Berk M . The effects of vitamin and mineral supplementation on symptoms of schizophrenia: a systematic review and meta-analysis. Psychol Med. (2017) 47:1515–27. doi: 10.1017/S0033291717000022 28202095

[156] Brown HE Roffman JL. Vitamin supplementation in the treatment of schizophrenia. CNS Drugs. (2014) 28:611–22. doi: 10.1007/s40263-014-0172-4 24846474 PMC4083629

[157] Arvindakshan M Ghate M Ranjekar PK Evans DR Mahadik SP. Supplementation with a combination of ω-3 fatty acids and antioxidants (vitamins E and C) improves the outcome of schizophrenia. Schizophr Res. (2003) 62:284. doi: 10.1016/S0920-9964(02)00284-0 12837515

[158] Valipour G Saneei P Esmaillzadeh A. Serum vitamin D levels in relation to schizophrenia: a systematic review and meta-analysis of observational studies. J Clin Endocrinol Metab. (2014) 99:1887. doi: 10.1210/jc.2014-1887 25050991

[159] Gurusamy J Gandhi S Damodharan D Ganesan V Palaniappan M. Exercise, diet and educational interventions for metabolic syndrome in persons with schizophrenia: a systematic review. Asian J Psychiatr. (2018) 36:73–85. doi: 10.1016/j.ajp.2018.06.018 29990631

[160] Bergman RN Kim SP Hsu IR Catalano KJ Chiu JD Kabir M . Abdominal obesity: role in the pathophysiology of metabolic disease and cardiovascular risk. Am J Med. (2007) 120:12. doi: 10.1016/j.amjmed.2006.11.012 17296343

[161] Phaniendra A Jestadi DB Periyasamy L. Free radicals: properties, sources, targets, and their implication in various diseases. Indian J Clin Biochem. (2015) 30:11–26. doi: 10.1007/s12291-014-0446-0 25646037 PMC4310837

[162] Young G Conquer J. Omega-3 fatty acids and neuropsychiatric disorders. Reprod Nutr Dev. (2005) 45:1–28. doi: 10.1051/rnd:2005001 15865053

[163] Rotariu D Babes EE Tit DM Moisi M Bustea C Stoicescu M . Oxidative stress – complex pathological issues concerning the hallmark of cardiovascular and metabolic disorders. Biomed Pharmacother. (2022) 152:113238. doi: 10.1016/j.biopha.2022.113238 35687909

[164] Bitanihirwe BKY Woo TUW. Oxidative stress in schizophrenia: an integrated approach. Neurosci Biobehav Rev. (2011) 35:878–93. doi: 10.1016/j.neubiorev.2010.10.008 20974172 PMC3021756

[165] Yao JK Keshavan MS. Antioxidants, redox signaling, and pathophysiology in schizophrenia: an integrative view. Antioxid Redox Signal. (2011) 15:2011–35. doi: 10.1089/ars.2010.3603 21126177 PMC3159108

[166] Ndhlala AR Moyo M Van Staden J. Natural antioxidants: fascinating or mythical biomolecules? Molecules. (2010) 15:905. doi: 10.3390/molecules15106905 PMC6259562 20938402

[167] Bentsen H Osnes K Refsum H Solberg DK Bøhmer T. A randomized placebo-controlled trial of an omega-3 fatty acid and vitamins E+C in schizophrenia. Transl. Psychiatry. (2013):3. doi: 10.1038/tp.2013.110 24346133 PMC3906471

[168] Ciobica A Padurariu M Dobrin I Stefanescu C Dobrin R. Oxidative stress in schizophrenia - focusing on the main markers. Psychiatr Danub. (2011) 23:237–45. PMID: 21963690

[169] Da Silva T Wu A Laksono I Prce I Maheandiran M Kiang M . Mitochondrial function in individuals at clinical high risk for psychosis. Sci Rep. (2018) 8:1–10. doi: 10.1038/s41598-018-24355-6 29670128 PMC5906614

[170] Roberts RC. Mitochondrial dysfunction in schizophrenia: with a focus on postmortem studies. Mitochondrion. (2020) 56:91. doi: 10.1016/J.MITO.2020.11.009 33221354 PMC7810242

[171] Ni P Chung S. Mitochondrial dysfunction in schizophrenia. BioEssays. (2020) 42:1900202. doi: 10.1002/BIES.201900202 32338416

[172] Murray AJ Rogers JC MZUH K Liddle PF Upthegrove R. Oxidative stress and the pathophysiology and symptom profile of schizophrenia Spectrum disorders. Front Psych. (2021) 12:703452. doi: 10.3389/fpsyt.2021.703452 PMC8339376 34366935

[173] Cuenod M Steullet P Cabungcal JH Dwir D Khadimallah I Klauser P . Caught in vicious circles: a perspective on dynamic feed-forward loops driving oxidative stress in schizophrenia. Mol Psychiatry. (2021) 27:1886–97. doi: 10.1038/s41380-021-01374-w 34759358 PMC9126811

[174] Rambaud V Marzo A Chaumette B. Oxidative stress and emergence of psychosis. Antioxidants MDPI. (2022) 11:1870. doi: 10.3390/ANTIOX11101870/S1 PMC9598314 36290593

[175] Murray AJ Rogers JC Katshu MZUH Liddle PF Upthegrove R. Oxidative stress and the pathophysiology and symptom profile of schizophrenia Spectrum disorders. Front Psychiatry. (2021) 12:703452. doi: 10.3389/FPSYT.2021.703452/BIBTEX 34366935 PMC8339376

[176] Ermakov EA Dmitrieva EM Parshukova DA Kazantseva DV Vasilieva AR Smirnova LP. Oxidative stress-related mechanisms in schizophrenia pathogenesis and new treatment perspectives. Oxid med cell Longev. (2021) 2021:8881770. doi: 10.1155/2021/8881770 33552387 PMC7847339

[177] Guan X Chen Y Wang X Xiu M Wu F Zhang X. Total antioxidant capacity, obesity and clinical correlates in first-episode and drug-naïve patients with schizophrenia. Schizophr Res. (2024):264. doi: 10.1016/j.schres.2023.12.004 38113675

[178] Müller N Weidinger E Leitner B Schwarz MJ. The role of inflammation in schizophrenia. Front Neurosci. (2015) 9:372. doi: 10.3389/fnins.2015.00372 26539073 PMC4612505

[179] Innes JK Calder PC. Omega-6 fatty acids and inflammation. Prostaglandins Leukotrienes Essent Fatty Acids. (2018) 132:41–8. doi: 10.1016/j.plefa.2018.03.004 29610056

[180] Miller BJ Goldsmith DR. Inflammatory biomarkers in schizophrenia: implications for heterogeneity and neurobiology. Biomarkers Neuropsychiatry. (2019) 1:100006. doi: 10.1016/j.bionps.2019.100006

[181] Li S Zhuo M Huang X Huang Y Zhou J Xiong D . Altered gut microbiota associated with symptom severity in schizophrenia. PeerJ. (2020):8. doi: 10.7717/peerj.9574 PMC7395597 32821537

[182] Yang C Lin X Wang X Liu H Huang J Wang S. The schizophrenia and gut microbiota: a bibliometric and visual analysis. Front Psychiatry. (2022) 13:1022472. doi: 10.3389/FPSYT.2022.1022472/BIBTEX 36458121 PMC9705344

[183] Christos T Maria-Ioana Stefanou Marina Demetriou Evangelor Alevyzakis Konstantinos Triantafyllou Spandidos Demetrios A . *Association of gut dysbiosis with first-episode psychosis*. (2024). Available at: https://www.spandidos-publications.com/10.3892/mmr.2024.13254?text=fulltext (Accessed Nov 2, 2024).

[184] Zheng P Zeng B Liu M Chen J Pan J Han Y . The gut microbiome from patients with schizophrenia modulates the glutamate-glutamine-GABA cycle and schizophrenia-relevant behaviors in mice. Sci Adv. American association for the. Adv Sci. (2019) 5:eaau8317. doi: 10.1126/SCIADV.AAU8317 PMC6365110 30775438

[185] Zhou K Baranova A Cao H Sun J Zhang F. Gut microbiome and schizophrenia: insights from two-sample Mendelian randomization. Schizophrenia. (2024) 10:1–6. doi: 10.1038/s41537-024-00497-7 39223235 PMC11369294

[186] Ju S Shin Y Han S Kwon J Choi TG Kang I . The gut–brain Axis in schizophrenia: the implications of the gut microbiome and SCFA production. Nutrients. (2023) 15:4391. doi: 10.3390/NU15204391 37892465 PMC10610543

[187] Deledda A Annunziata G Tenore GC Palmas V Manzin A Velluzzi F. Diet-derived antioxidants and their role in inflammation, obesity and gut microbiota modulation. Antioxidants. (2021) 10:708. doi: 10.3390/antiox10050708 33946864 PMC8146040

[188] Pet MA Brouwer-Brolsma EM. The impact of maternal vitamin D status on offspring brain development and function: A systematic review. Adv Nutr. (2016) 7:665–78. doi: 10.3945/an.115.010330 27422502 PMC4942857

[189] Debnath M Venkatasubramanian G Berk M. Fetal programming of schizophrenia: select mechanisms. Neurosci Biobehav Rev. (2015) 49:90–104. doi: 10.1016/j.neubiorev.2014.12.003, PMID: 25496904 PMC7112550

[190] Baker GB Hallak JEC Dilullo AF Burback L Dursun SM. Amino acids in schizophrenia – Glycine, serine and arginine In: Baker GB, editor. Handbook of Schizophrenia Spectrum Disorders, vol. 1. Berlin: Springer (2011)

[191] Zhang Y Yin J Yan H Yan L Li Y Zhang C . Correlations between omega-3 fatty acids and inflammatory/glial abnormalities: the involvement of the membrane and neurotransmitter dysfunction in schizophrenia. Front Cell Neurosci. (2023):17. doi: 10.3389/fncel.2023.1163764 PMC10626455 37937262

[192] Bostock ECS Kirkby KC Taylor BVM. The current status of the ketogenic diet in psychiatry. Front Psych. (2017) 8:43. doi: 10.3389/fpsyt.2017.00043 PMC5357645 28373848

[193] Ntalkitsi S Efthymiou D Bozikas V Vassilopoulou E. Halting the metabolic complications of antipsychotic medication in patients with a first episode of psychosis: how far can we go with the Mediterranean diet? A pilot study. Nutrients. (2022) 14:5012. doi: 10.3390/nu14235012 36501042 PMC9738803

[194] Teasdale SB Ward PB Rosenbaum S Watkins A Curtis J Kalucy M . A nutrition intervention is effective in improving dietary components linked to cardiometabolic risk in youth with first-episode psychosis. Br J Nutr. (2016) 115:1033. doi: 10.1017/S0007114516001033 27153205

[195] Zhang L Zhu M Liu X Zhao Z Han P Lv L . Calorie-restricted diet mitigates weight gain and metabolic abnormalities in obese women with schizophrenia: a randomized controlled trial. Front Nutr. (2023) 1:10. doi: 10.3389/fnut.2023.1038070 PMC10198382 37215202

[196] Redman LM Ravussin E. Caloric restriction in humans: impact on physiological, psychological, and behavioral outcomes. Antioxid Redox Signal. (2011) 14:275–87. doi: 10.1089/ars.2010.3253 20518700 PMC3014770

[197] Zubrzycki A Cierpka-Kmiec K Kmiec Z Wronska A. The role of low-calorie diets and intermittent fasting in the treatment of obesity and type-2 diabetes. J Physiol Pharmacol. (2018) 69:2. doi: 10.26402/jpp.2018.5.02 30683819

[198] Leidy HJ Clifton PM Astrup A Wycherley TP Westerterp-Plantenga MS Luscombe-Marsh ND . The role of protein in weight loss and maintenance. Am J Clin Nutr. (2015) 101:1320S–9S. doi: 10.3945/ajcn.114.084038 25926512

[199] Dickerson F Gennusa JV Stallings C Origoni A Katsafanas E Sweeney K . Protein intake is associated with cognitive functioning in individuals with psychiatric disorders. Psychiatry Res. (2020):284. doi: 10.1016/j.psychres.2019.112700 31791705

[200] Buosi P Borghi FA Lopes AM da Silva FI Fernandes-Ferreira R Oliveira-Brancati CIF . Oxidative stress biomarkers in treatment-responsive and treatment-resistant schizophrenia patients. Trends Psychiatry Psychother. (2021) 43:78. doi: 10.47626/2237-6089-2020-0078 PMC8835384 34982515

[201] Ben Othmen L Mechri A Fendri C Bost M Chazot G Gaha L . Altered antioxidant defense system in clinically stable patients with schizophrenia and their unaffected siblings. Prog Neuro-Psychopharmacol Biol Psychiatry. (2008) 32:3. doi: 10.1016/j.pnpbp.2007.08.003 17804133

[202] Reyazuddin M Azmi SA Islam N Rizvi A. Oxidative stress and level of antioxidant enzymes in drug-naive schizophrenics. Indian J Psychiatry. (2014) 56:516. doi: 10.4103/0019-5545.146516 PMC4279291 25568474

[203] Djordjevic V Lazarevic D Cosic V Knezevic M Djordjevic V. Age-related changes of superoxide dismutase activity in patients with schizophrenia. Vojnosanit Pregl. (2017) 74:142. doi: 10.2298/vsp141202142d 29350504

[204] Djordjević VV Kostić J Krivokapić Ž Krtinić D Ranković M Petković M . Decreased activity of erythrocyte catalase and glutathione peroxidase in patients with schizophrenia. Medicina (Lithuania). (2022) 58:1491. doi: 10.3390/medicina58101491 PMC9609318 36295651

[205] Gawryluk JW Wang JF Andreazza AC Shao L Young LT. Decreased levels of glutathione, the major brain antioxidant, in post-mortem prefrontal cortex from patients with psychiatric disorders. Int J Neuropsychopharmacol. (2011) 14:805. doi: 10.1017/S1461145710000805 20633320

[206] Yao JK Leonard S Reddy R. Altered glutathione redox state in schizophrenia. Dis Markers. (2006) 22:387. doi: 10.1155/2006/248387 PMC3850558 16410648

[207] Dietrich-Muszalska A Kwiatkowska A. Generation of superoxide anion radicals and platelet glutathione peroxidase activity in patients with schizophrenia. Neuropsychiatr Dis Treat. (2014) 10:703–9. doi: 10.2147/NDT.S60034 24833903 PMC4015795

[208] Michel TM Thome J Martin D Nara K Zwerina S Tatschner T . Cu, Zn- and Mn-superoxide dismutase levels in brains of patients with schizophrenic psychosis. J Neural Transm. (2004) 111:160. doi: 10.1007/s00702-004-0160-9 15338334

[209] Abdalla DSP Monteiro HP Oliveira JAC Bechara EJH. Activities of superoxide dismutase and glutathione peroxidase in schizophrenic and manic-depressive patients. Clin Chem. (1986) 32:805. doi: 10.1093/clinchem/32.5.805 2870827

[210] Djordjević V. Superoxide dismutase in psychiatric diseases. London: IntechOpen (2022).

[211] Hurşitoğlu O Orhan FÖ Kurutaş EB Doğaner A Durmuş HT Kopar H. Diagnostic performance of increased malondialdehyde level and oxidative stress in patients with schizophrenia. Noropsikiyatri Arsivi. (2021) 58:372. doi: 10.29399/npa.27372 PMC8419726 34526839

[212] Rukmini MS D’Souza B D’Souza V. Superoxide dismutase and catalase activities and their correlation with malondialdehyde in schizophrenic patients. Indian J Clin Biochem. (2004) 19:114–8. doi: 10.1007/BF02894268 23105467 PMC3454217

[213] Sarandol A Kirli S Akkaya C Altin A Demirci M Sarandol E. Oxidative-antioxidative systems and their relation with serum S100 B levels in patients with schizophrenia: effects of short term antipsychotic treatment. Prog Neuro-Psychopharmacol Biol Psychiatry. (2007) 31:8. doi: 10.1016/j.pnpbp.2007.03.008 17459548

[214] Surapaneni K Venkataramana G. Status of lipid peroxidation, glutathione, ascorbic acid, vitamin E and antioxidant enzymes in patients with osteoarthritis. Indian J Med Sci. (2007) 61:592. doi: 10.4103/0019-5359.29592 17197733

[215] Wu Z Zhang XY Wang H Tang W Xia Y Zhang FX . Elevated plasma superoxide dismutase in first-episode and drug naive patients with schizophrenia: inverse association with positive symptoms. Prog Neuro-Psychopharmacol Biol Psychiatry. (2012) 36:18. doi: 10.1016/j.pnpbp.2011.08.018 21896300

[216] Gong Y Zhao R Yang B. Superoxide dismutase activity and malondialdehyde levels in patients with travel-induced psychosis. Shanghai arch. Psychiatry. (2012) 24:5. doi: 10.3969/j.issn.1002-0829.2012.03.005 PMC4198847 25324620

[217] Li XR Xiu MH Guan XN Wang YC Wang J Leung E . Altered antioxidant defenses in drug-naive first episode patients with schizophrenia are associated with poor treatment response to risperidone: 12-week results from a prospective longitudinal study. Neurotherapeutics. (2021) 18:36. doi: 10.1007/s13311-021-01036-3 PMC8423973 33791970

[218] Do KQ Bovet P Cuenod M. Schizophrenia: glutathione deficit as a new vulnerability factor for disconnectivity syndrome. Schweizer Archiv Neurol Psychiatr. (2004) 155:1534. doi: 10.4414/sanp.2004.01534

[219] Raffa M Atig F Mhalla A Kerkeni A Mechri A. Decreased glutathione levels and impaired antioxidant enzyme activities in drug-naive first-episode schizophrenic patients. BMC Psychiatry. (2011) 11:124. doi: 10.1186/1471-244X-11-124 21810251 PMC3161936

[220] Langbein K Hesse J Gussew A Milleit B Lavoie S Amminger GP . Disturbed glutathione antioxidative defense is associated with structural brain changes in neuroleptic-naïve first-episode psychosis patients. Prostaglandins Leukot Essent Fatty Acids. (2018) 136:5. doi: 10.1016/j.plefa.2017.10.005 29111383

[221] Palaniyappan L Park MTM Jeon P Limongi R Yang K Sawa A . Is there a glutathione centered redox dysregulation subtype of schizophrenia? Antioxidants. (2021) 10:1703. doi: 10.3390/antiox10111703 34829575 PMC8615159

[222] Iwata Y Nakajima S Plitman E Truong P Bani-Fatemi A Caravaggio F . Glutathione levels and glutathione-glutamate correlation in patients with treatment-resistant schizophrenia. Schizophr Bull Open. (2021) 2:6. doi: 10.1093/schizbullopen/sgab006 PMC8086698 33969302

[223] Gunes M Camkurt MA Demir S Ibiloglu A Kaya MC Bulut M . Serum malonyldialdehyde levels of patients with schizophrenia. Klin Psikofarmakol Bul. (2015) 25:S109–10.

[224] Kropp S Kern V Lange K Degner D Hajak G Kornhuber J . Oxidative stress during treatment with first- and second-generation antipsychotics. J Neuropsychiatry Clin Neurosci. (2005) 17:227. doi: 10.1176/jnp.17.2.227 15939978

[225] Uddin SMN Sultana F Uddin MG Dewan SMR Hossain MK Islam MS. Effect of antioxidant, malondialdehyde, macro-mineral, and trace element serum concentrations in Bangladeshi patients with schizophrenia: a case-control study. Health Sci Rep. (2021) 4:291. doi: 10.1002/hsr2.291 PMC8112814 34013069

[226] Xiang YZ Yun LT Dong FZ Lian YC Gui YW Haile CN . Disrupted antioxidant enzyme activity and elevated lipid peroxidation products in schizophrenic patients with tardive dyskinesia. J Clin Psychiatry. (2007) 68:513. doi: 10.4088/jcp.v68n0513 17503985

[227] Dakhale GN Khanzode SD Khanzode SS Saoji A. Supplementation of vitamin C with atypical antipsychotics reduces oxidative stress and improves the outcome of schizophrenia. Psychopharmacology. (2005) 182:117. doi: 10.1007/s00213-005-0117-1 16133138

[228] Sarandol A Sarandol E Acikgoz HE Eker SS Akkaya C Dirican M. First-episode psychosis is associated with oxidative stress: effects of short-term antipsychotic treatment. Psychiatry Clin Neurosci. (2015) 69:1233. doi: 10.1111/pcn.12333 26172069

[229] Al-Fartusie FS Al-Bairmani HK Al-Garawi ZS Yousif AH. Evaluation of some trace elements and vitamins in major depressive disorder patients: a case–control study. Biol Trace Elem Res. (2019) 189:1507. doi: 10.1007/s12011-018-1507-7 30238421

[230] Myken AN Ebdrup BH Sørensen ME Broberg BV Skjerbæk MW Glenthøj BY . Lower vitamin C levels are associated with less improvement in negative symptoms in initially antipsychotic-Naïve patients with first-episode psychosis. Int J Neuropsychopharmacol. (2022) 25:29. doi: 10.1093/ijnp/pyac029 PMC9380709 35532335

[231] Subotičanec K Folnegović-Šmalc V Korbar M Meštrović B Buzina R. Vitamin C status in chronic schizophrenia. Biol Psychiatry. (1990) 28:61. doi: 10.1016/0006-3223(90)90061-6 2275953

[232] Sampson TR Debelius JW Thron T Janssen S Shastri GG. Gut microbiota regulate motor deficits and neuroinflammation in a model of Parkinson’s disease. Cell. (2021) 184:143–160. 10.1016/j.cell.2016.11.018 PMC5718049 27912057

